# Synergism Between Controlled-Release Fertilization and Microbial Bioinputs Modulates the Morphophysiological Quality of *Prunus* Rootstock Genotypes

**DOI:** 10.1007/s00284-026-04793-6

**Published:** 2026-03-03

**Authors:** João Antônio Paraginski, Mariana Poll Moraes, Newton Alex Mayer, Valmor João Bianchi

**Affiliations:** 1 Departamento de Fitotecnia, Universidade Federal de Pelotas (UFPel), Campus Capão do Leão. Capão do Leão, Rio Grande do Sul, Brasil; 2 Embrapa Clima Temperado. Pelotas, Rio Grande do Sul, Brasil; 3 Departamento de Botânica, Universidade Federal de Pelotas (UFPel), Campus Capão do Leão. Capão do Leão, Rio Grande do Sul, Brasil

## Abstract

The production of high-quality *Prunus* seedling rootstocks in soilless systems requires optimizing the interaction between genetics, nutrient availability, and rhizosphere microbiology. This study evaluated the morphophysiological response of four peach rootstock genotypes (‘Capdeboscq’, “Okinawa Roxo”, “NR0060408”, and “NR0160305”) to inoculation with *Trichoderma asperellum* and a microbial consortium (*Bacillus amyloliquefaciens* + *Trichoderma harzianum*), under contrasting doses of controlled-release fertilizer (0 and 4 g dm^− 3^ of CRF). Longitudinal analysis (0-120 days) demonstrated that nutrient availability is the primary limiting factor; microbial bioinputs did not compensate for the absence of fertilization due to the metabolic cost of symbiosis. However, under nutrient sufficiency (4 g dm^− 3^), strong synergism was observed. The new selections (“NR0060408” and “NR0160305”) exhibited high phenotypic plasticity, maximizing the conversion of biostimulation into shoot biomass and outperforming the “Okinawa Roxo” genotype, which displayed a conservative growth strategy. The *Bacillus*-*Trichoderma* consortium was superior to single inoculation in responsive genotypes, potentiating seedling leaf area and height. Furthermore, inoculation promoted the “stay-green” effect, maintaining chlorophyll index stability until the end of the cycle. It is concluded that the use of bioinputs, especially in a consortium, acts as a metabolic catalyst in responsive genotypes, but their efficacy depends on adequate basal nutritional management.

**Supplementary Information:**

The online version contains supplementary material available at 10.1007/s00284-026-04793-6.

## Introduction

Peach production [*Prunus persica* (L.) Batsch] in Brazil is concentrated primarily in the South and Southeast regions, with the states of Rio Grande do Sul and São Paulo as the main producers [1]. Although the country ranks among the top 20 global producers, the average national yield (13.37 Mg ha^− 1^) is considered low compared to world benchmarks such as Jordan (27.78 Mg ha^− 1^) and Chile (21.89 Mg ha^− 1^) [2]. This low yield, particularly in Rio Grande do Sul (11.97 Mg ha^− 1^) [1], is frequently associated with the quality of propagation material, seedling production, and traditional nursery systems [3–6].

Historically, in southern Brazil, rootstocks have been produced from seeds obtained from pits discarded by the canning industry [7], generating highly heterozygous material [6–8]. This varietal mixture results in high genetic and vigor variability, leading to uneven development patterns and variable tolerance to biotic and abiotic stresses [9–12]. Consequently, the use of genotypes selected for this purpose is essential, meeting criteria for stability [9], growth uniformity, and nutritional efficiency [8, 13]. In this context, the search for improved genotypes for rootstock function must be continuous, especially due to the limited number of *Prunus* rootstock cultivars registered in Brazil [14]. Although scion cultivars such as ‘Capdeboscq’ and ‘Aldrighi’ remain as traditional references [15, 16], current alternatives include ‘Okinawa’, ‘Flordaguard’, and selections such as “Okinawa Roxo” [8, 10, 17, 18]. Such materials stand out for important traits such as vigor, nematode resistance [4, 19, 20], and, fundamentally, their specific nutritional demands throughout the growth phase [17, 21–23].

Despite genetic advances, comprehensive literature on the nutritional requirements, physiological responses, and growth patterns of these genotypes in modern containerized seedling production systems is still incipient in Brazil [17, 21, 24, 25]. This gap is significant, given that Brazilian liming and fertilization manuals [26, 27] focus on soil management in established orchards, rather than on specific nutritional practices for the rootstock production phase and seedlings in nurseries. Therefore, it is crucial to establish optimal nutritional ranges that optimize growth and increase nutrient absorption efficiency [21, 22, 24]. Recent studies seek to determine nutrient doses that potentiate uptake and positively impact photosynthetic pigment levels, such as chlorophyll, influencing growth and dry matter partitioning [13, 21–24, 28–30].

Aligned with the pursuit of greater nutritional efficiency, the use of beneficial microorganisms emerges as a complementary strategy. Species of the genus *Trichoderma* (*Trichoderma* spp.) are widely studied for their dual function as biocontrol agents and, crucially, as plant growth promoters [31, 32]. Fungal interactions result in biomass gains [32] and improvements in morphological attributes [24], due to the capacity of *Trichoderma* spp. to promote ion uptake (mitigating genetic variations in absorption), optimize carbohydrate metabolism, and mediate phytohormone synthesis [31, 33, 34]. The maximum efficacy of *Trichoderma* spp. is, however, intrinsically linked to nutrient availability in the rhizosphere environment [35]. Adequate ranges of Nitrogen, Phosphorus, Potassium, and micronutrients are fundamental to optimize colonization, enzymatic activity, and phytohormone synthesis promoted by the fungus [32, 36, 37]. This dependence justifies the integration of *Trichoderma* into controlled nutritional management. In the Brazilian context, *T. asperellum* is widely recommended for its proven efficacy as a plant growth promoter and biocontrol agent in soil and substrate [37, 38]. Previous results in *P. persica* [24] have already indicated that the *T. asperellum* strain CCT 2165 can increase chlorophyll content and growth in rootstocks, reinforcing its viability. Complementarily, inoculation strategies can explore the synergistic potential of microbial consortia, such as the combination of *T. harzianum* and *Bacillus amyloliquefaciens*. This interaction is based on complementary modes of action: while *B. amyloliquefaciens* stimulates growth via indole-3-acetic acid (IAA) production, mobilizes nutrients (favoring phosphate and zinc solubilization), and acts in phytopathogen suppression [39], *T. harzianum* acts as a robust development modulator, promoting increased IAA and gibberellins (GA) in *Prunus*, which results in better root and shoot structuring [40]. Therefore, the co-inoculation of these microorganisms could theoretically outperform single-strain applications by simultaneously optimizing nutrient availability in the soil solution and the plant’s capacity to intercept these resources.

Although the individual and synergistic potential of these microorganisms is recognized, the literature is incipient regarding studies evaluating the performance of different peach rootstock genotypes under the triple interaction of genotype, controlled fertilization (different CRF doses), and the inoculation of these microorganisms (*Trichoderma asperellum*; *Bacillus amyloliquefaciens* + *T. harzianum*) via commercial bioinputs. Similarly, the need to test promising materials such as peach rootstocks is even more evident due to the low number of rootstocks registered in Brazil, which restricts management options in nurseries and requires the search for more efficient alternatives. Thus, understanding this genotype-nutrition-biological interaction is essential to optimize nursery protocols and ensure high-quality seedlings. In this context, the tested hypotheses were: (i) The morphophysiological performance of rootstocks will be modulated by the triple interaction, with new rootstock selections (“NR0060408” and “NR0160305”) presenting the best indicators, outperforming traditional genotypes, especially under nutritional sufficiency (4 g dm^− 3^ of CRF) combined with inoculation; and (ii) The application of the bioinput based on *B. amyloliquefaciens* + *T. harzianum*, due to their synergistic mechanisms (IAA production and nutrient solubilization), will result in growth and chlorophyll content increments superior to inoculation with *T. asperellum* and the *Control* treatment (without microorganism inoculation). Therefore, the objective of this work was to evaluate the morphophysiological response (growth and chlorophyll indices) of four peach rootstock genotypes (*Prunus persica* L. Batsch) to inoculation with bioinputs based on *T. asperellum* and *B. amyloliquefaciens* + *T. harzianum*, under different controlled fertilization scenarios (0 and 4 g dm^− 3^ of CRF).

## Materials and Methods

### Experimental Material

The experiment was conducted between September 2023 and February 2024 at the Universidade Federal de Pelotas (UFPel) Campus Capão do Leão (31º48’03” S, 52º24’41” W). Peach (*Prunus persica*) rootstocks propagated by seeds from the cultivar Capdeboscq and selections Okinawa Roxo, NR0060408 (‘Capdeboscq’ × ‘Nemaguard’), and NR0160305 (‘Nemaguard’ × ‘Capdeboscq’) were used. Selections NR0060408 and NR0160305 were obtained through controlled crosses performed at UFPel between peach rootstocks with the aim of developing genetic materials adapted to the edaphoclimatic conditions of Southern Brazil and resistant to nematodes, aiming at expanding commercial areas and increasing seedling production efficiency. The choice of these materials considered attributes relevant to nursery performance, such as growth uniformity and efficient nutrient uptake capacity, as reported by Menegatti et al. [17], Souza et al. [21], and Paraginski et al. [24, 25].

Ripe fruits were harvested manually between January 10 and February 15, 2023, from mother plants belonging to the *Prunus* spp. rootstock germplasm collection of UFPel, maintained under field conditions and located at 31º48’08” S, 52º30’24” W, at an altitude of 40 m. After harvest, pits were extracted manually, washed in running water, and dried in the shade for 30 days. Subsequently, they were stored in Kraft paper bags under refrigeration at 4 ± 2 °C until the beginning of the stratification process.

In May 2023, the pits were individually cracked using a bench vise for seed extraction, which were placed in Petri dishes (90 × 15 mm) containing two sheets of germination paper moistened with 2 mL of fungicide solution (1.5 g L^− 1^ of Sialex^®^ 500 + 2.5 g L^− 1^ of Orthocide^®^ 500). The seeds remained under stratification for 80 days under refrigeration (4 ± 2 °C) in the dark.

After the stratification period, germinated seeds were sown in 72-cell polystyrene trays (114 cm^3^ per cell) containing commercial substrate Carolina Soil^®^, composed of *Sphagnum* peat, expanded vermiculite, dolomitic limestone, agricultural gypsum, and traces of N-P-K fertilizer. Additionally, the substrate possessed the following characteristics: pH = 5.5 ± 0.5; Electrical conductivity = 0.7 ± 0.3 mS cm^− 1^; Density = 145 kg m^− 3^; Water holding capacity = 55.0%. To complement nutritional availability, the substrate was supplemented with 4 g dm^− 3^ of controlled-release fertilizer Osmocote^®^ 14-14-14 (N-P-K) and 4 g dm^− 3^ of dolomitic agricultural limestone (CaO = 26.50%; Mg = 15.00%; Total relative neutralizing power = 76.16%).

When seedlings reached approximately 20 cm in height, they were transplanted into perforated plastic bags with a capacity of 1.7 dm^3^, filled with the aforementioned commercial substrate, supplemented exclusively with 4 g dm^− 3^ of dolomitic agricultural limestone, aiming to adjust the substrate pH to values close to 6.0, a range considered suitable for the development of peach rootstocks. This standardization aimed to ensure substrate chemical stability and allow for the precise application of nutritional and biological treatments throughout the experiment.

## Experimental Design

The experiment was conducted in a completely randomized design, in a 4 × 2 × 3 factorial arrangement. The factors included four peach rootstock genotypes (‘Capdeboscq’, “Okinawa Roxo”, “NR0060408”, “NR0160305”), two doses of controlled-release fertilizer (0 and 4 g dm^− 3^ of CRF), and three bioinput treatments (No Bioinput = *Control*, Tríppel^®^ = *T. asperellum*, Torpeno^®^ = *B. amyloliquefaciens* + *T. harzianum*), totaling 24 treatments, with five replicates of one plant each. Detailed information regarding the experimental matrix, as well as the chemical and biological specifications of the inputs, is summarized in Table 1. For the fertilized treatments, the CRF (Basacote^®^ Plus 9 M) was homogeneously incorporated into the substrate before transplanting the rootstocks into the plastic bags.

**Table 1:** Experimental matrix and technical characterization of the nutritional and biological inputs used in the production of peach rootstocks

I. Experimental design^a^			
Genotypes	‘Capdeboscq’, “Okinawa Roxo”, “NR0060408”, “NR0160305”		4 levels of genetic material
CRF Doses	0 and 4 g dm^− 3^ (Basacote^®^ Plus 9 M)		2 nutritional scenarios
Bioinputs	*Control* (No Bioinput), Tríppel^®^, Torpeno^®^		3 microbial management levels
II. Chemical Composition			
Macronutrients (%)		*N* = 16.0; P_2_O_5_ = 8.0; K_2_O = 12.0; S = 5.0; Mg = 1.2	
Micronutrients (%)		B = 0.02; Cu: 0.05; Fe = 0.40; Mn = 0.06; Mo = 0.015; Zn = 0.02	
III. Biological specs			
Bioinput 1 (Tríppel^®^)		*Trichoderma asperellum* (CCT 2165) – 1 × 10^5^ CFU mL^− 1^	
Bioinput 2 (Torpeno^®^)		*B. amyloliquefaciens* (CPQBA 040-11DRM 01/04) – 1 × 10^7^ CFU g^− 1^; *T. harzianum* (CPQBA 040-11DRM 09) – 1 × 10^7^ CFU g^− 1^	

^a^The combination of factors resulted in 24 distinct treatments (e.g., T1: ‘Capdeboscq’ + 0 g dm^-3^ + *Control*; T24: “NR0160305” + 4 g dm^-3^ + Torpeno^®^). CFU: Colony Forming Units. All bioinputs were applied at 0 and 40 days after transplanting

The selection of 0 and 4 g dm^− 3^ CRF doses aimed to evaluate bioinput performance in two contrasting scenarios: a substrate without fertilization, to verify the microorganisms’ capacity to promote plant growth in the absence of additional nutrients; and another with adequate nutrient supply, allowing the analysis of plant response to inoculation under conditions of adequate nutritional availability. The 4 g dm^− 3^ dose was selected based on previous studies indicating positive growth responses of peach rootstocks under CRF fertilization.

*T. asperellum* inoculation was performed by applying a solution prepared with 4 mL of distilled water for each 1 mL of the commercial product Tríppel^®^ (strain CCT 2165; 1 × 10^5^ CFU mL^− 1^). The co-inoculation of *B. amyloliquefaciens* and *T. harzianum* was performed using the commercial product Torpeno^®^ [*B. amyloliquefaciens* (strains CPQBA 040-11DRM 01 and 04; 1 × 10^7^ CFU g^− 1^) + *T. harzianum* (strain CPQBA 040 − 11 DRM 09; 1 × 10^7^ CFU g^− 1^)], diluted at a ratio of 5 mL of distilled water for each 0.01 g of the product. Applications were performed directly onto the substrate at two moments: on the day of transplanting (0 DAT) and 40 days after transplanting (40 DAT), always between 17:00 and 19:00 h. Application doses were 5 mL dm^− 3^ at 0 DAT and 10 mL dm^− 3^ at 40 DAT.

## Analyzed Variables

Experimental evaluations included plant height (PH, cm), measured from the base to the plant apex using a tape measure; stem diameter (SD, mm), measured at 10 cm from the plant base using a digital caliper; number of leaves (NL, leaves plant^− 1^); leaf area (LA, cm^2^), estimated according to Sachet et al. [41]; and chlorophyll *a* (Chl *a*), *b* (Chl *b*), and *a + b* (Chl *a + b*) contents, determined in FCI units (Falker Chlorophyll Index) using a Falker^®^ chlorophyll meter (model ClorofiLOG – CLF1030), on leaves from the middle third of the plants. Variables were measured at 0, 20, 40, 60, 80, 100, and 120 DAT to monitor plant growth and evaluate treatment effects. Non-destructive methods were employed exclusively throughout the experimental period to preserve rootstock integrity for a subsequent study focused on nutritional partitioning and grafting efficiency. The final evaluation, at 120 DAT, was used for detailed analysis, as it represented the stage of greatest differentiation between treatments and because fertilized plants had reached the minimum diameter (5.00 mm) suitable for grafting. The remaining evaluations, performed at periodic intervals, were fundamental to ensure uniform growth trends among treatments and to assist in understanding the final results.

### Statistical Analysis

**Fig. 1:**
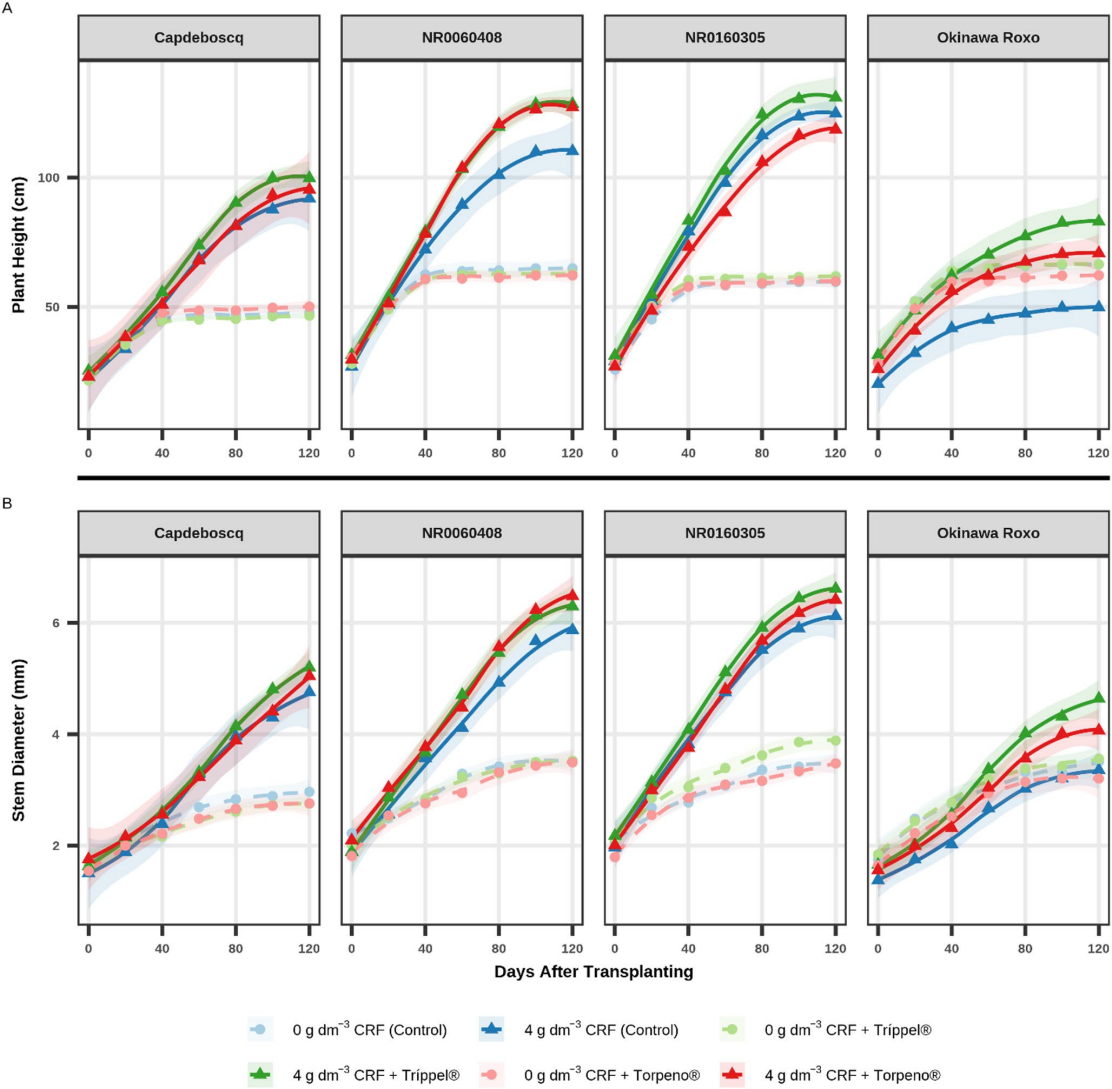
Linear growth trajectory: (**A**) Plant height (PH, cm); and (**B**) Stem diameter (SD, mm) of four peach rootstock genotypes over 120 days after transplanting (DAT). Panels facet the genotypes. Points represent treatment means at each evaluation date to enhance visual clarity, while lines indicate LOESS smoothing curves (shaded 95% confidence interval) fitted using the full raw dataset to preserve statistical rigor. The unified legend identifies the six treatments: circles (●) and dashed lines represent 0 g dm^− 3^ CRF; triangles (▲) and solid lines represent 4 g dm^− 3^ CRF

**Fig. 2:**
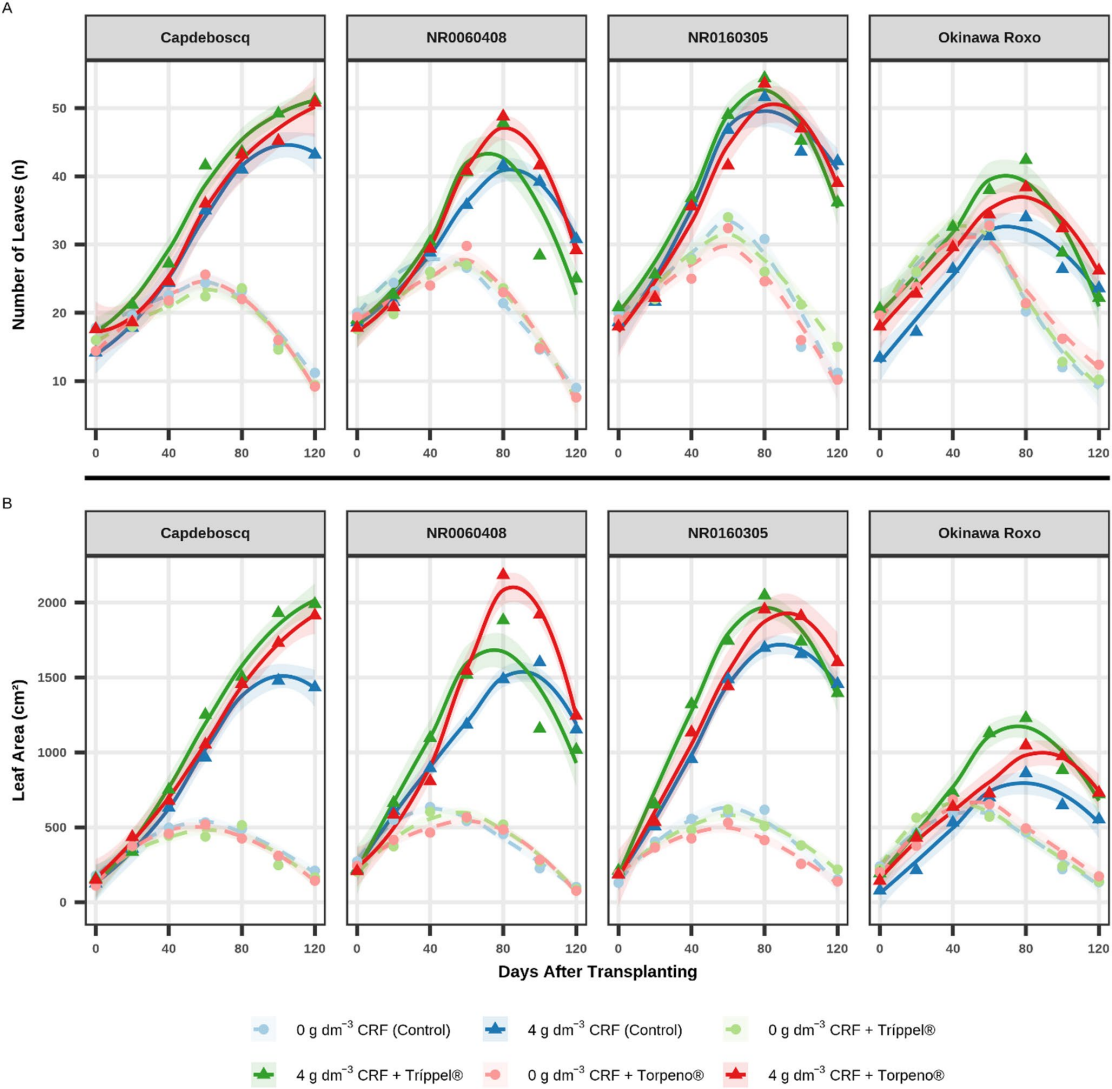
Development of foliar biometric variables: (**A**) Number of leaves (NL, n), and; (**B**) Leaf area (LA, cm^2^) of four peach rootstock genotypes during 120 days after transplanting (DAT). Panels facet the genotypes. Points represent treatment means at each evaluation date to enhance visual clarity, while lines indicate LOESS smoothing curves (shaded 95% confidence interval) fitted using the full raw dataset to preserve statistical rigor. The unified legend identifies the six treatments: circles (●) and dashed lines represent 0 g dm^− 3^ CRF; triangles (▲) and solid lines represent 4 g dm^− 3^ CRF

**Fig. 3:**
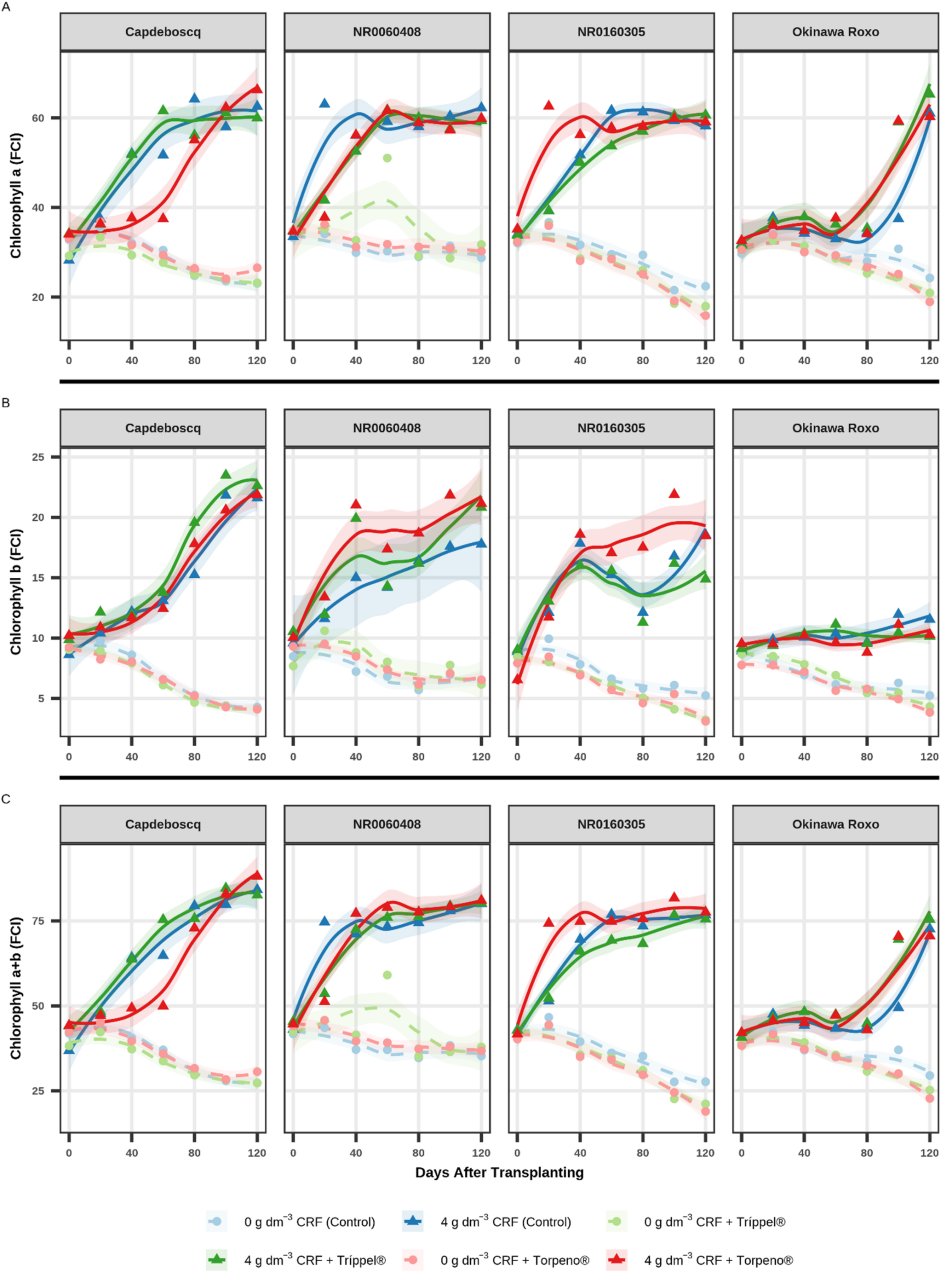
Temporal dynamics of chlorophyll indices (Falker Index - FCI): (**A**) Chlorophyll *a* (Chl *a*); (**B**) Chlorophyll *b* (Chl *b*), and; (**C**) Total Chlorophyll (Chl *a + b*) of four peach rootstock genotypes during 120 days after transplanting (DAT). Panels facet the genotypes. Points represent treatment means at each evaluation date to enhance visual clarity, while lines indicate LOESS smoothing curves (shaded 95% confidence interval) fitted using the full raw dataset to preserve statistical rigor. The unified legend identifies the six treatments: circles (●) and dashed lines represent 0 g dm^− 3^ CRF; triangles (▲) and solid lines represent 4 g dm^− 3^ CRF

All data were analyzed using R software v4.5.0 [42]. The experimental design was completely randomized in a 4 × 2 × 3 factorial arrangement, comprising four rootstock genotypes (G), two controlled-release fertilizer doses (C), and three bioinput treatments (B), with five replicates. The significance level (*α*) adopted for all statistical tests was 0.05. Data preparation was performed using the *tidyverse* package [43]. To meet the assumptions of generalized models, count variables (Number of Leaves, NL) were converted to integers (*NF_int*). Positive continuous variables presenting zero values (Plant Height, Leaf Area, Chlorophylls) were corrected by adding a minimum value (0.001), allowing the fitting of models with Gamma distribution. For linear growth and physiological trajectories (Figs. 1 and 2, and 3), data points represent the treatment means at each evaluation time (0 to 120 DAT) to enhance visual clarity, while LOESS smoothing curves were fitted using the full raw dataset to preserve statistical confidence intervals. The statistical analysis was divided into three main approaches:

1) Longitudinal Analysis (0–120 DAT) with Mixed Models (LMM/GLMM)

To evaluate the plant development trajectory over time (0, 20, 40, 60, 80, 100, and 120 DAT), Linear Mixed Models (LMM) or Generalized Linear Mixed Models (GLMM) were fitted using the *lme4* package [44]. This approach was chosen for its ability to model the dependence structure of repeated measures on the same experimental unit. The selection of error distribution and link function was specific to each response variable: Stem Diameter (SD) – LMM with Gaussian distribution (identity link function), via *lmer* function; Number of Leaves (NL) – GLMM with Negative Binomial distribution (log link function), via *glmer.nb* function, to correct for overdispersion (variance greater than the mean) in count data; Other Variables (PH, LA, Chl *a*, *b*, *a + b*) – GLMM with Gamma distribution (log link function), via *glmer* function, suitable for skewed positive continuous data.

The full mathematical model included the fixed effects of Genotype (G), CRF (C), Bioinput (B), the continuous effect of Time (T, Days After Transplanting), and all their interactions. Time was modeled as a second-degree polynomial (*T + T*^*2*^) to capture non-linear growth patterns. The unique plant identification (*Plant_ID*) was included as a random intercept effect (*1/Planta_ID*). The general model can be described as:

$$\:g\left(E\left[{Y}_{ijklm}\right]\right)={\eta}_{ijklm}+u_m$$

where: 𝑔(·) is the link function (logarithmic for Gamma/Negative Binomial; identity for Gaussian); $$\:{\eta\:}_{ijklm}$$ is the linear predictor of fixed effects, modeled as: $$\:\eta\:={G}_{i}+{C}_{j}+{B}_{k}+\left(T+{T}^{2}\right)+{\left(G\times\:C\right)}_{ij}+$$$$\:\cdot \cdot \cdot+(G\times\:C\times\:B\times\:(T+{T}^{2}){)}_{ijkl}$$ ; and u_m_ is the random intercept effect for plant *m*, with $$u_m \sim N\left(0,{\sigma\:}_{p}^{2}\right)$$.

The significance of fixed effects was evaluated using Analysis of Deviance (Type III ANOVA, χ^2^ Wald tests) using the *Anova* function of the *car* package [45]. For the unfolding of significant interactions with time, instantaneous growth rates (slopes) were estimated and compared at the experiment midpoint (60 DAT), using the *emtrends* function of the *emmeans* package [46]. Pairwise comparisons (adjusted by the Tukey test) were performed for: (1) the effect of Bioinputs within each G × C, and (2) the effect of Genotypes within each C × B.

2) Cross-sectional Analysis (120 DAT) with Generalized Models (GLM)

To compare treatments at the experiment endpoint (120 DAT), Linear Models (LM) or Generalized Linear Models (GLM) were fitted. Distribution selection was: SD – Linear Model (LM/ANOVA) with Gaussian distribution (*aov* function); NL – GLM with Poisson distribution (log link function); Other Variables (PH, LA, Chl *a*, *b*, *a + b*) – GLM with Gamma distribution (log link function). Effect significance was evaluated with Type III ANOVA (F-test for LM; χ^*2*^ Wald tests for GLMs) (*car* package). Due to the significance of the triple interaction G × C × B, a double *post-hoc* unfolding was performed. Estimated marginal means (EMMs) (*emmeans* package) were compared using the Tukey HSD test (via *multcomp* package [47]) to: 1 – Evaluate the effect of Bioinputs (B) within each Genotype × CRF combination; 2 – Evaluate the effect of Genotypes (G) within each CRF × Bioinput combination.

3) Multivariate Analysis (PCA) and Supplementary Data

To visualize the correlation structure between variables and treatment clustering at 120 DAT, a Principal Component Analysis (PCA) was performed based on the correlation matrix (standardized data, *scale.unit = TRUE*), using the *FactoMineR* package [48]. The results were visualized through a composite biplot, where individual scores for each genotype were faceted in separate panels, and variable loadings (vectors) were displayed in an optimized sub-graph to avoid label overlap and improve interpretability. Additionally, descriptive mean tables (± standard deviation) for all variables at all time points were calculated and formatted as supplementary material.

## Results

### Longitudinal Treatment Effects (0–120 DAT)

The temporal evaluation of growth variables (Figs. 1A and B and 2A, and B) and chlorophyll indices (Fig. 3A and B, and C) revealed distinct response patterns, primarily influenced by the CRF dose.

Treatments supplied with 4 g dm^− 3^ of CRF (solid lines) demonstrated markedly superior development compared to treatments without fertilization (0 g dm^− 3^ CRF, dashed lines). This divergence in growth became evident from 60 DAT for Plant Height (PH, Fig. 1A) and Stem Diameter (SD, Fig. 1B), and as early as 40 DAT for Number of Leaves (NL, Fig. 2A) and Leaf Area (LA, Fig. 2B).

A similar pattern was observed for chlorophyll indices (Fig. 3A and B, and C). Rootstocks grown with 4 g dm^− 3^ of CRF maintained or increased their Chlorophyll *a*, *b*, and *a + b* contents throughout the cycle, indicating nutritional sufficiency. In contrast, treatments without CRF presented a clear declining trend from 80 to 100 DAT, suggesting nutrient depletion in the substrate and the onset of leaf senescence.

The Analysis of Variance (Type III ANOVA) of the Longitudinal Mixed Models (GLMM) confirmed that time (DAT), CRF dose, and Genotype, as well as their interactions, were the most significant sources of variation (*p* < 0.05) (Table S2). The significance of high-order interactions (e.g., G × C × B × DAT) validated the analysis of growth rates (slopes) and the cross-sectional analysis at the endpoint.

Comparisons of instantaneous growth rates (slopes), estimated at the experiment midpoint (60 DAT), revealed that the most significant differences in growth “velocity” (vigor) occurred among bioinputs in CRF treatments and among genotypes under different management scenarios. Detailed statistical results of these slope comparisons are presented in the Supplementary Material (Tables S3 and S4).

## Cross-Sectional Analysis (120 DAT): Bioinput Effect

The analysis of variance at the experiment endpoint (120 DAT) (Table 2) confirmed the existence of significant triple interactions (G × C × B) for key variables, including PH (*p* = 0.0266), LA (*p* < 0.0001), and chlorophyll indices (*p* < 0.0008). This statistical significance required the unfolding of interactions, the results of which are presented below.

**Table 2:** Summary of Analysis of Variance (Type III ANOVA) for the effects of Genotype, CRF, Bioinput factors, and their interactions on growth and chlorophyll variables at 120 DAT

Source of Variation	df	PH			SD			NL			LA		
		Statistic	*p*-value		Statistic		*p*-value	Statistic	*p*-value		Statistic		*p*-value
Genotype (G)	3	19.4909	0.0002		2.0043		0.1185	1.7451	0.6270		48.5417		0.0000
CRF (C)	1	41.4277	0.0000		76.5280		0.0000	63.1183	0.0000		431.4357		0.0000
Bioinput (B)	2	0.2980	0.8616		0.0073		0.9928	0.7956	0.6718		7.1726		0.0277
G × C	3	98.3790	0.0000		21.9771		0.0000	5.1173	0.1634		52.5056		0.0000
G × B	6	2.0976	0.9105		0.6245		0.7103	8.4245	0.2086		40.7351		0.0000
C × B	2	3.4410	0.1790		1.4734		0.2343	0.3943	0.8211		7.1550		0.0279
G × C × B	6	14.2871	0.0266		0.8856		0.5087	8.7029	0.1910		31.2834		0.0000

Source of Variation	df	Chl *a*				Chl *b*				Chl *a + b*			
		Statistic		p-value		Statistic		p-value		Statistic		p-value	
Genotype (G)	1	18.7767		0.0003		25.9273		0.0000		25.2088		0.0000	
CRF (C)	2	142.3727		0.0000		144.1407		0.0000		193.3598		0.0000	
Bioinput (B)	3	2.3779		0.3045		0.6390		0.7265		1.5536		0.4599	
G × C	6	7.0572		0.0701		56.1938		0.0000		15.8762		0.0012	
G × B	2	43.7938		0.0000		32.9174		0.0000		53.3134		0.0000	
C × B	6	2.6161		0.2703		3.6848		0.1584		0.7179		0.6984	
G × C × B	3	23.1310		0.0008		10.3440		0.1109		22.9642		0.0008	

Summary of Type III Analysis of Variance (ANOVA) at 120 DAT. The Statistic value refers to the F-test for the SD variable (Gaussian model) and the Wald χ^2^ test for all other variables (Gamma or Poisson GLM models). The significance (p < 0.05) of the interactions justifies the unfolding of the Tukey test presented in Tables 3 and 4.

The Table 3 details the effect of bioinputs (*Control*, Tríppel^®^, Torpeno^®^) within each genotype and CRF dose combination. In treatments without CRF (0 g dm^− 3^), the bioinput effect was generally subtle, with few statistical differences for PH, SD, and NL (Table 3). However, a significant effect was observed for Leaf Area (LA), where for ‘Capdeboscq’ and “NR0060408”, the *Control* treatment resulted in higher LA (210.67 cm^2^ and 102.24 cm^2^, respectively) compared to the Torpeno^®^ treatment.

The bioinput effect was more pronounced at the 4 g dm^− 3^ CRF dose (Table 3). For “Okinawa Roxo”, the application of Tríppel^®^ (83.00 cm) and Torpeno^®^ (70.80 cm) resulted in taller plants (PH) than the *Control* (49.80 cm). For ‘Capdeboscq’ and “Okinawa Roxo”, bioinput treatments (Tríppel^®^ and Torpeno^®^) also led to greater Leaf Area (LA) compared to the *Control*.

For chlorophyll indices (Table 3), a distinct pattern emerged in treatments without CRF (0 g dm^− 3^): *Control* treatments frequently presented chlorophyll *a* and *a + b* indices superior to bioinput treatments, especially in genotypes “NR0160305” and “Okinawa Roxo”. However, at the 4 g dm^− 3^ CRF dose, these differences were mostly non-significant, suggesting that controlled-release fertilization masked the differential effects of bioinputs on foliar pigments at the end of the cycle.

**Table 3:** Growth variables (PH, SD, NL, LA) and chlorophyll (Chl *a*, *b*, *a + b*) of peach rootstocks at 120 DAT, in response to CRF doses (0 and 4 g dm^− 3^) and bioinputs (*Control*, Tríppel^®^, Torpeno^®^)

Variable	CRF	Bioinput	‘Capdeboscq’	“Okinawa Roxo”	“NR0060408”	“NR0160305”
PH (cm)	0 g dm^− 3^	*Control*	47.76 a	66.40 a	64.90 a	59.70 a
		Tríppel^®^	46.60 a	66.50 a	62.80 a	61.84 a
		Torpeno^®^	50.12 a	62.20 a	62.20 a	59.90 a
	4 g dm^− 3^	*Control*	91.96 a	49.80 a	110.20 a	124.80 a
		Tríppel^®^	99.80 a	83.00 b	128.40 a	131.00 a
		Torpeno^®^	95.34 a	70.80 b	127.20 a	118.60 a
SD (mm)	0 g dm^− 3^	*Control*	2.97 a	3.51 a	3.52 a	3.48 a
		Tríppel^®^	2.75 a	3.56 a	3.52 a	3.89 a
		Torpeno^®^	2.76 a	3.20 a	3.49 a	3.48 a
	4 g dm^− 3^	*Control*	4.76 a	3.36 b	5.87 a	6.12 a
		Tríppel^®^	5.20 a	4.64 a	6.30 a	6.61 a
		Torpeno^®^	5.05 a	4.07 a	6.48 a	6.41 a
NL (n)	0 g dm^− 3^	*Control*	11.20 a	9.80 a	9.00 a	11.20 a
		Tríppel^®^	9.40 a	10.20 a	7.60 a	15.00 a
		Torpeno^®^	9.20 a	12.40 a	7.60 a	10.20 a
	4 g dm^− 3^	*Control*	43.20 a	23.60 a	30.80 a	42.20 a
		Tríppel^®^	51.20 a	22.20 a	25.00 a	36.20 a
		Torpeno^®^	50.80 a	26.20 a	29.20 a	39.00 a
LA (cm^2^)	0 g dm^− 3^	*Control*	210.67 a	134.31 b	102.24 a	152.47 b
		Tríppel^®^	165.53 ab	140.70 ab	87.86 ab	218.37 a
		Torpeno^®^	143.95 b	173.20 a	77.06 b	139.52 b
	4 g dm^− 3^	*Control*	1433.61 b	553.01 b	1152.89 a	1457.19 a
		Tríppel^®^	1990.06 a	720.97 a	1017.51 a	1395.73 a
		Torpeno^®^	1913.85 a	731.24 a	1244.66 a	1602.83 a
Chl *a* (FCI)	0 g dm^− 3^	*Control*	23.06 a	24.26 a	28.76 a	22.42 a
		Tríppel^®^	23.18 a	20.92 ab	31.74 a	17.96 b
		Torpeno^®^	26.54 a	18.94 b	30.24 a	15.86 b
	4 g dm^− 3^	*Control*	62.56 a	61.08 a	62.24 a	58.18 a
		Tríppel^®^	59.98 a	65.28 a	59.42 a	60.64 a
		Torpeno^®^	66.26 a	60.28 a	59.86 a	59.10 a
Chl *b* (FCI)	0 g dm^− 3^	*Control*	4.28 a	5.24 a	6.50 a	5.24 a
		Tríppel^®^	4.12 a	4.32 ab	6.16 a	3.22 b
		Torpeno^®^	4.10 a	3.84 b	6.54 a	3.10 b
	4 g dm^− 3^	*Control*	21.60 a	11.54 a	17.78 a	18.54 a
		Tríppel^®^	22.62 a	10.14 a	20.82 a	14.88 b
		Torpeno^®^	21.86 a	10.28 a	21.14 a	18.46 a
Chl *a + b* (FCI)	0 g dm^− 3^	*Control*	27.34 a	29.50 a	35.26 a	27.66 a
		Tríppel^®^	27.30 a	25.24 b	37.90 a	21.18 b
		Torpeno^®^	30.64 a	22.78 b	36.78 a	18.96 b
	4 g dm^− 3^	*Control*	84.16 a	72.62 a	80.02 a	76.72 a
		Tríppel^®^	82.60 a	75.42 a	80.24 a	75.52 a
		Torpeno^®^	88.12 a	70.56 a	81.00 a	77.56 a

^a^Means followed by the same letter within the same column (Genotype) do not differ statistically by Tukey’s HSD test (p > 0.05). The test evaluates the effect of bioinputs (*Control*, Tríppel^®^, Torpeno^®^) independently for each CRF level (0 and 4 g dm^-3^), within each Genotype. Grouping letters for the 0 g dm^-3^ level are independent of letters for the 4 g dm^-3^ level

## Cross-Sectional Analysis (120 DAT): Genotype Performance

The Table 4 presents the reverse unfolding, comparing the performance of the four genotypes within each management scenario (CRF × Bioinput). A strong Genotype × CRF interaction was observed (Table 2). In the absence of CRF (0 g dm^− 3^, “Okinawa Roxo” and “NR0060408” were, in general, the tallest genotypes (PH), while ‘Capdeboscq’ consistently presented the lowest height. For Leaf Area (LA), “NR0060408” recorded the lowest values (77.06 cm^2^ with Torpeno^®^ and 87.86 cm^2^ with Tríppel^®^), while ‘Capdeboscq’ and “NR0160305” (with Tríppel^®^) showed the highest LA.

The performance scenario changed drastically with the addition of CRF (4 g dm^− 3^). Genotypes “NR0160305” and “NR0060408” showed greater growth under high nutritional availability, reaching the highest PH and SD means (e.g., “NR0160305” with 4 g dm^− 3^ CRF + Tríppel^®^ reached 131.00 cm PH and 6.61 mm SD; “NR0060408” with 4 g dm^− 3^ CRF + Torpeno^®^ reached 127.20 cm PH and 6.48 mm SD). Notably, “Okinawa Roxo”, which performed well without CRF, presented the most restricted growth in PH and SD under the 4 g dm^− 3^ CRF dose, especially in the Control treatment (49.80 cm and 3.36 mm, respectively). Genotype ‘Capdeboscq’ presented the highest Leaf Area (LA) value when combined with bioinputs (e.g., 1990.06 cm^2^ with 4 g dm^− 3^ CRF + Tríppel^®^) (Table 4).

For chlorophyll indices (Table 4), “NR0060408” stood out in treatments without CRF, presenting the highest Chl *a + b* contents (e.g., 37.90 FCI with 0 g dm^− 3^ + Tríppel^®^), while “NR0160305” showed the lowest indices in this scenario. With 4 g dm^− 3^ of CRF, ‘Capdeboscq’ tended to accumulate more total chlorophyll (e.g., 88.12 FCI with 4 g dm^− 3^ CRF + Torpeno^®^), while “Okinawa Roxo” recorded the lowest values (e.g., 70.56 FCI).

**Table 4:** Comparison of growth variables (PH, SD, NL, LA) and chlorophyll (Chl *a*, *b*, *a + b*) of peach rootstocks at 120 DAT, under different CRF and bioinput management scenarios

Variables	Genotypes	0 g dm^− 3^ CRF + Control	0 g dm^− 3^ CRF + Tríppel^®^	0 g dm^− 3^ CRF + Torpeno^®^	4 g dm^− 3^ CRF + Control	4 g dm^− 3^ CRF + Tríppel^®^	4 g dm^− 3^ CRF + Torpeno^®^
PH (cm)	‘Capdeboscq’	47.76 b	46.60 b	50.12 b	91.96 b	99.80 b	95.34 b
	“Okinawa Roxo”	66.40 a	66.50 a	62.20 a	49.80 c	83.00 b	70.80 c
	“NR0060408”	64.90 a	62.80 a	62.20 a	110.20 ab	128.40 a	127.20 a
	“NR0160305”	59.70 a	61.84 a	59.90 ab	124.80 a	131.00 a	118.60 a
SD (mm)	‘Capdeboscq’	2.97 a	2.75 b	2.76 b	4.76 b	5.20 b	5.05 b
	“Okinawa Roxo”	3.51 a	3.56 a	3.20 ab	3.36 c	4.64 b	4.07 c
	“NR0060408”	3.52 a	3.52 a	3.49 a	5.87 a	6.30 a	6.48 a
	“NR0160305”	3.48 a	3.89 a	3.48 a	6.12 a	6.61 a	6.41 a
NL (n)	‘Capdeboscq’	11.20 a	9.40 ab	9.20 a	43.20 a	51.20 a	50.80 a
	“Okinawa Roxo”	9.80 a	10.20 ab	12.40 a	23.60 b	22.20 c	26.20 c
	“NR0060408”	9.00 a	7.60 b	7.60 a	30.80 b	25.00 c	29.20 c
	“NR0160305”	11.20 a	15.00 a	10.20 a	42.20 a	36.20 b	39.00 b
LA (cm²)	‘Capdeboscq’	210.67 a	165.53 b	143.95 a	1433.61 a	1990.06 a	1913.85 a
	“Okinawa Roxo”	134.31 bc	140.70 b	173.20 a	553.01 b	720.97 d	731.24 c
	“NR0060408”	102.24 c	87.86 c	77.06 b	1152.89 a	1017.51 c	1244.66 b
	“NR0160305”	152.47 b	218.37 a	139.52 a	1457.19 a	1395.73 b	1602.83 ab
Chl *a* (FCI)	‘Capdeboscq’	23.06 b	23.18 b	26.54 a	62.56 a	59.98 a	66.26 a
	“Okinawa Roxo”	24.26 b	20.92 bc	18.94 b	61.08 a	65.28 a	60.28 a
	“NR0060408”	28.76 a	31.74 a	30.24 a	62.24 a	59.42 a	59.86 a
	“NR0160305”	22.42 b	17.96 c	15.86 c	58.18 a	60.64 a	59.10 a
Chl *b* (FCI)	‘Capdeboscq’	4.28 b	4.12 b	4.10 b	21.60 a	22.62 a	21.86 a
	“Okinawa Roxo”	5.24 b	4.32 b	3.84 bc	11.54 b	10.14 c	10.28 b
	“NR0060408”	6.50 a	6.16 a	6.54 a	17.78 a	20.82 a	21.14 a
	“NR0160305”	5.24 b	3.22 c	3.10 c	18.54 a	14.88 b	18.46 a
Chl *a + b* (FCI)	‘Capdeboscq’	27.34 b	27.30 b	30.64 b	84.16 a	82.60 a	88.12 a
	“Okinawa Roxo”	29.50 b	25.24 b	22.78 c	72.62 a	75.42 a	70.56 b
	“NR0060408”	35.26 a	37.90 a	36.78 a	80.02 a	80.24 a	81.00 ab
	“NR0160305”	27.66 b	21.18 c	18.96 d	76.72 a	75.52 a	77.56 ab

^a^Means followed by the same letter within the same column (Management) do not differ statistically by the Tukey HSD test (p > 0.05). The test evaluates the performance of the four genotypes (rows) independently for each CRF and Bioinput combination (columns)

^a^ Means followed by the same letter within the same column (Management) do not differ statistically by the Tukey HSD test (*p* > 0.05). The test evaluates the performance of the four genotypes (rows) independently for each CRF and Bioinput combination (columns).

### Multivariate Analysis (PCA)

**Fig. 4:**
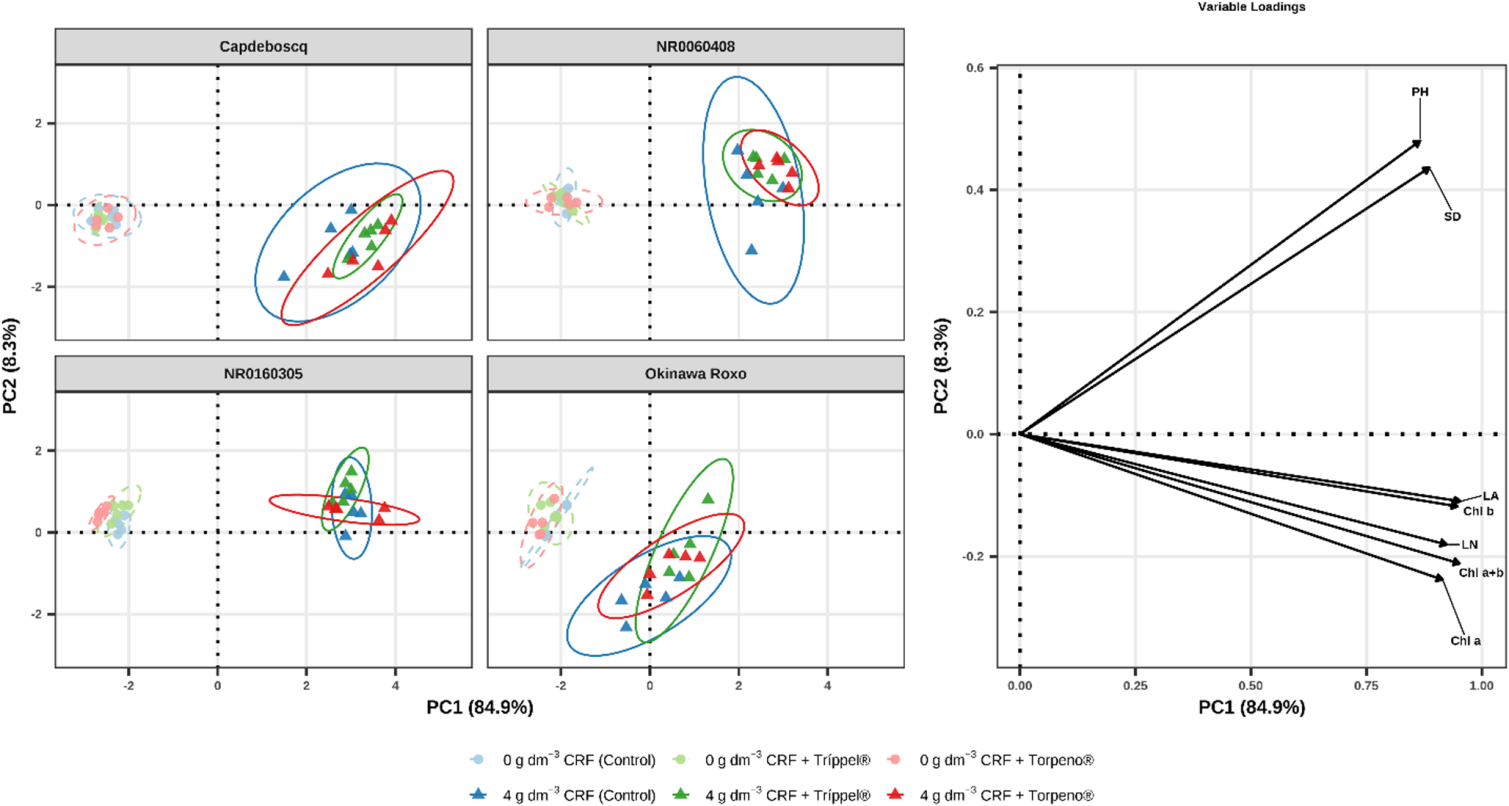
Composite biplot of the Principal Component Analysis (PCA) integrating growth and physiological variables at 120 days after transplanting (DAT). The four panels on the left display individual scores for each genotype (points represent individual observations and ellipses the 95% confidence interval). The sub-graph on the right (Variable Loadings) displays the correlation vectors (black arrows) with optimized label positioning (PH, SD, LN, LA, Chl *a*, *b*, *a + b*) to avoid overlap and improve interpretability. Axis titles (PC1 and PC2) are highlighted in bold with their respective explained variance percentage. The unified legend identifies the six treatments

Principal Component Analysis (PCA) synthesized the total data variation at 120 DAT and confirmed the observed patterns (Fig. 4). The first principal component (PC1) was the main separation vector, explaining 84.9% of the total variation. PC1 was strongly positively correlated with all growth (PH, SD, NL, LA) and chlorophyll (Chl *a*, *b*, *a + b*) variables. This axis unequivocally separated treatments with 4 g dm^− 3^ of CRF (grouped to the right, with positive scores on PC1) from those with 0 g dm^− 3^ (grouped to the left, with negative scores), confirming that fertilization was the factor with the greatest impact on plant development.

The second component (PC2), explaining 8.3% of the variation, assisted in differentiating between genotypes and bioinput effects. For example, in the “Okinawa Roxo” panel, the ellipse of the *Control* treatment (4 g dm^− 3^ of CRF) is clearly separated (negative scores on PC2) from the ellipses of Tríppel^®^ and Torpeno^®^ (positive scores on PC2), indicating a distinct multifactorial response to bioinput application in this genotype.

## Discussion

The clear separation observed between 0 and 4 g dm^− 3^ CRF doses (PC1, Fig. 4) corroborates that nutrient availability is the primary limiting factor for the development of containerized peach rootstocks, initially overshadowing biological effects. The absence of significant gains in height and stem diameter at the 0 g dm^− 3^ dose (Table 3) aligns with the findings of Bononi et al. [35] and Halifu et al. [36]. These authors report that colonization efficacy and microbial metabolic activity in the rhizosphere, processes involving extracellular enzyme synthesis and phytohormone production, are energetically costly and dependent on a basal level of fertility. Without minimal nutritional support, the metabolic cost of maintaining symbiosis may drain photoassimilate reserves, limiting the plant’s immediate vegetative response [32, 37]. On the other hand, the positive response observed at the 4 g dm^− 3^ dose, especially for leaf area and shoot vigor, confirms the hypothesis of synergism between mineral nutrition and biostimulation. In this sufficiency scenario, the gradual nutrient release by CRF allows the plant to allocate resources to feed the microbiota, which reciprocates by potentiating root uptake through rhizosphere acidification and increased plasma membrane H^+^-ATPase activity [31, 38].

The positive response observed at the 4 g dm^− 3^ dose confirms the hypothesis of synergism between mineral nutrition and biostimulation. This behavior aligns with previous findings where Menegatti and Bianchi [49] observed superior growth of cultivar ‘Capdeboscq’ up to 8 g dm^− 3^, while Souza et al. [21] reported high fertilization efficiency for “Okinawa Roxo”. Furthermore, our results expand the observations of Paraginski et al. [24], who identified the 4 to 6 g dm^− 3^ CRF range as ideal for peach rootstocks and reported that *T. asperellum* inoculation could optimize fertilization efficiency, even allowing for dose reductions of up to 1.34 g dm^− 3^ without compromising growth. In the present work, it is fundamental to highlight that, as characterized by Paraginski et al. [25], selections “NR0060408” and “NR0160305” possess superior initial vigor compared to the standard cultivar ‘Capdeboscq’. This high genetic growth capacity justifies the elevated nutritional demand of these genotypes and explains the expressive response to biostimulation at the 4 g dm^− 3^ dose, where the Torpeno^®^ consortium maximized available resource use efficiency [17, 21]. Ultimately, this approach identifies whether microorganisms act as growth promoters in nutritional limitation scenarios or contribute to additive gains when nutrients are at adequate levels.

The superiority of the bioinput Torpeno^®^ (consortium: *T. harzianum* + *B. amyloliquefaciens*) over Tríppel^®^ (isolate: *T. asperellum*) in responsive genotypes (Table 4) validates the occurrence of complementary mechanisms operating in distinct functional niches. While *Trichoderma* species present in both bioinputs act predominantly on the physical and hormonal modulation of root architecture via auxin and gibberellin signaling [34, 40], *B. amyloliquefaciens* (exclusive to Torpeno^®^) performs a specialized biochemical role. This rhizobacteria stands out for biofilm formation and the secretion of organic acids and phosphatases into the soil solution, essential mechanisms for the solubilization of recalcitrant P and Zn fractions [39]. The coexistence of these microorganisms creates an interception synergism: the expansion of soil exploration surface promoted by the fungus maximizes the uptake of nutrients chemically mobilized by the bacterium. This functional coupling is particularly advantageous in the controlled-release fertilization system, as it allows for the interception of ions released by granules before losses via leaching occur. Furthermore, the division of metabolic tasks within the consortium may reduce the individual energy cost for the plant compared to maintaining a single symbiosis under high demand, channeling the carbon surplus to leaf area expansion [33]. This gain in shoot biomass is a critical, yet necessary, energetic sink to ensure the production of rootstocks with ideal diameter and reserves for grafting [9, 15].

The maintenance of high chlorophyll *a*, *b*, and *a + b* indices (FCI) until 120 DAT in fertilized and inoculated treatments reflects a superior nutritional status and the manifestation of the functional stay-green phenomenon. The positive correlation observed between chlorophyll indices and the continuous biomass increment indicates that the photosynthetic machinery remained active throughout the experimental period in treatments fertilized with 4 g dm^− 3^ of CRF. Physiologically, this delay in senescence can be attributed to hormonal modulation induced by bioinputs: both *Trichoderma* spp. and *B. amyloliquefaciens* are capable of stimulating the endogenous synthesis of cytokinins and auxins, signaling the plant to maintain chloroplast integrity [34, 40]. In parallel, Huang et al. [28] and Bononi et al. [35] highlight that Mg and Fe availability, mediated by solubilizing microorganisms, is essential for the stability of the chlorophyll molecule. Our results corroborate and expand upon the findings of Paraginski et al. [24] who observed increments in photosynthetic efficiency in rootstocks inoculated with *T. asperellum*. The stability of pigments under a high growth rate suggests that bioinputs assisted in Nitrogen homeostasis, preventing the early remobilization of basal leaves. This effect is crucial, as root confinement in containers with reduced volume acts as a classic abiotic stress, accelerating leaf senescence by limiting the pool of available nutrients [22, 30]. From an agronomic point of view, this prolongation of photosynthetic activity maximizes the accumulation of carbohydrate reserves, which are fundamental for grafting success [15].

Multivariate analysis revealed that “bio-responsiveness” is a complex genetic trait, regulated by sink strength and the intrinsic efficiency of each genotype [8]. Selections “NR0160305” and “NR0060408” demonstrated high phenotypic plasticity, responding aggressively to the CRF + Bioinput combination (Table 4). This behavior is consistent with the initial characterization performed by Paraginski et al. [25], who identified a germination potential superior to the standard cultivar ‘Capdeboscq’ in these selections, indicating a genetic predisposition for rapid establishment. Physiologically, this response suggests that “NR” genotypes possess a high capacity to convert the nutritional surplus mobilized by microorganisms into biomass. This corroborates the findings of Paraginski et al. [24] with the related selection “NR0170302”, where inoculation optimized nutrient use efficiency. In the present study, the addition of *B. amyloliquefaciens* in the Torpeno^®^ consortium amplified this efficiency, acting as a metabolic catalyst for elite genotypes [12]. In contrast, “Okinawa Roxo” exhibited a conservative strategy: it presented robust performance under low fertility but reached a growth plateau at the high dose. This indicates that its adaptation involves high acquisition efficiency under limiting conditions, but lower utilization efficiency for rapid growth under nutritional luxury [21, 23]. Therefore, inoculation acts distinctly according to the target: maximizing productive potential in responsive genotypes and promoting resilience in rustic genotypes.

Despite the progress in understanding the interaction between rootstock genotypes, mineral fertilization, and bioinputs, this study presents limitations that should be acknowledged. The primary constraint involves the absence of analytical data on the mineral composition of plant tissues, which precluded a direct assessment of potential variations in nutrient uptake and accumulation efficiency across the different treatments. This methodological decision was part of a research strategy to decouple the comprehensive morphophysiological characterization presented here from a subsequent study focused exclusively on the nutritional dynamics of these rootstocks. Regarding future perspectives, data from grafting experiments with scion cultivars are currently being processed to identify how the rootstock/scion combination modulates nutrient partitioning. Furthermore, ongoing trials are evaluating the morphology and mineral partitioning of grafted seedlings inoculated with the Torpeno^®^ consortium, aiming to consolidate biostimulation as a practical management tool to optimize seedling quality in the nursery phase.

## Conclusion

Nutritional availability via controlled-release fertilizer is the determining factor for the viability of nursery biostimulation. Inoculation with *Trichoderma* and *Bacillus* does not replace basal fertilization but acts synergistically in the presence of nutritional sufficiency (4 g dm^− 3^), maximizing vegetative vigor and promoting the maintenance of photosynthetic pigments (stay-green) until the grafting stage.

Responsiveness to inoculation is a variable genetic trait. Selections “NR0160305” and “NR0060408” present high phenotypic plasticity, efficiently converting biostimulation into shoot biomass under adequate nutrition, while the “Okinawa Roxo” genotype exhibits a conservative strategy, with superior performance under low fertility but limited response to increased inputs.

The microbial consortium (*T. harzianum* + *B. amyloliquefaciens*) demonstrates superior efficacy compared to single inoculation (*T. asperellum*) in promoting leaf expansion and height growth in responsive genotypes, validating the functional complementarity between microorganisms as a strategy to optimize peach rootstock production in intensive cultivation systems.

## Supplementary Information

Below is the link to the electronic supplementary material.

Supplementary Material 1

## Data Availability

The datasets generated during and/or analyzed during the current study are available from the corresponding author on reasonable request.
