# Supplementary material for: Synergism Between Controlled-Release Fertilization and Microbial Bioinputs Modulates the Morphophysiological Quality of Prunus Rootstock Genotypes
Source: Curr Microbiol. 2026 Mar 3;83(4):220. doi: 10.1007/s00284-026-04793-6 (PMC12957629; doi:10.1007/s00284-026-04793-6)
Supplement: Supplementary file 1 — Supplementary Material 1 [file 284_2026_4793_MOESM1_ESM.docx]

**Synergism between controlled-release fertilization and microbial bioinputs modulates the morphophysiological quality of *Prunus* rootstock genotypes**

**João Antônio Paraginski**

Departamento de Fitotecnia, Universidade Federal de Pelotas (UFPel) Campus Capão do Leão. Capão do Leão, Rio Grande do Sul, Brasil. [joaoantonioparaginski@gmail.com](mailto:joaoantonioparaginski@gmail.com), <https://orcid.org/0000-0003-0690-9402>;

**Mariana Poll Moraes**

Departamento de Fitotecnia, Universidade Federal de Pelotas (UFPel) Campus Capão do Leão. Capão do Leão, Rio Grande do Sul, Brasil. [maripollmoraes@gmail.com](mailto:maripollmoraes@gmail.com), <https://orcid.org/0000-0001-7546-7025>;

**Newton Alex Mayer**

Embrapa Clima Temperado. Pelotas, Rio Grande do Sul, Brasil. [alex.mayer@embrapa.br](mailto:alex.mayer@embrapa.br), <https://orcid.org/0000-0001-6689-8202>;

**Valmor João Bianchi**

Departamento de Botânica, Universidade Federal de Pelotas (UFPel) Campus Capão do Leão. Capão do Leão, Rio Grande do Sul, Brasil. [valmorjb@yahoo.com](mailto:valmorjb@yahoo.com), <https://orcid.org/0000-0002-7473-5523>.

**Corresponding Author:**

João Antônio Paraginski

Departamento de Fitotecnia, Universidade Federal de Pelotas (UFPel) Campus Capão do Leão. Capão do Leão, Rio Grande do Sul, Brasil. [joaoantonioparaginski@gmail.com](mailto:joaoantonioparaginski@gmail.com)

**Suplementary Data**

**Table S1.** Summary of Analysis of Deviance (Type III ANOVA) for the fixed effects of Longitudinal Mixed Models (LMM/GLMM) from 0-120 DAT, detailing all terms of the polynomial model.

| term | df | ----- PH ----- | | | ----- SD ----- | | | ----- NL ----- | | | | ----- LA ----- | | |
| --- | --- | --- | --- | --- | --- | --- | --- | --- | --- | --- | --- | --- | --- | --- |
|  |  | statistic | p.value | | statistic | | p.value | statistic | | p.value | | statistic | | p.value |
| (Intercept) | 1 | 1945.8084 | 0.0000 | | 161.9988 | | 0.0000 | 1183.6194 | | 0.0000 | | 9323.2240 | | 0.0000 |
| Genotype (G) | 3 | 8.5827 | 0.0354 | | 4.6282 | | 0.2011 | 2.7141 | | 0.4378 | | 80.8167 | | 0.0000 |
| CRF (C) | 1 | 0.9708 | 0.3245 | | 2.0117 | | 0.1561 | 1.9821 | | 0.1592 | | 5.1552 | | 0.0232 |
| Bioinput (B) | 2 | 0.5126 | 0.7739 | | 1.0722 | | 0.5850 | 3.1517 | | 0.2068 | | 25.5452 | | 0.0000 |
| DAT | 1 | 2824.8067 | 0.0000 | | 43.3708 | | 0.0000 | 22.3156 | | 0.0000 | | 3546.7495 | | 0.0000 |
| I(DAT^2^) | 1 | 3829.7260 | 0.0000 | | 10.7414 | | 0.0010 | 41.8038 | | 0.0000 | | 27903.7288 | | 0.0000 |
| G × C | 3 | 8.6644 | 0.0341 | | 1.2815 | | 0.7135 | 4.9734 | | 0.1813 | | 150.9713 | | 0.0000 |
| G × B | 6 | 2.3082 | 0.8893 | | 0.6216 | | 0.9960 | 2.8977 | | 0.8216 | | 55.7084 | | 0.0000 |
| C × B | 2 | 0.9929 | 0.6087 | | 2.3237 | | 0.3129 | 1.5756 | | 0.4548 | | 11.7768 | | 0.0028 |
| G × DAT | 3 | 17.1945 | 0.0006 | | 1.8800 | | 0.5977 | 3.2629 | | 0.3528 | | 622.4968 | | 0.0000 |
| G × I(DAT^2^) | 3 | 29.6523 | 0.0000 | | 0.6029 | | 0.8958 | 4.4684 | | 0.2151 | | 1187.7290 | | 0.0000 |
| C × DAT | 1 | 258.7415 | 0.0000 | | 24.2707 | | 0.0000 | 0.7898 | | 0.3741 | | 166.4666 | | 0.0000 |
| C × I(DAT^2^) | 1 | 127.7155 | 0.0000 | | 0.0121 | | 0.9125 | 3.2873 | | 0.0698 | | 1672.5553 | | 0.0000 |
| B × DAT | 2 | 30.4892 | 0.0000 | | 0.5954 | | 0.7425 | 1.5303 | | 0.4653 | | 243.0618 | | 0.0000 |
| B × I(DAT^2^) | 2 | 46.9100 | 0.0000 | | 0.2923 | | 0.8640 | 0.8702 | | 0.6472 | | 1066.5200 | | 0.0000 |
| G × C × B | 6 | 6.0575 | 0.4168 | | 0.8094 | | 0.9918 | 4.3273 | | 0.6325 | | 67.8156 | | 0.4168 |
| G × C × DAT | 3 | 253.6243 | 0.0000 | | 35.0280 | | 0.0000 | 0.7932 | | 0.9204 | | 431.9477 | | 0.0000 |
| G × C × I(DAT^2^) | 3 | 252.1367 | 0.0000 | | 11.6853 | | 0.0085 | 0.2043 | | 0.9769 | | 1381.7765 | | 0.0000 |
| G × B × DAT | 6 | 32.1071 | 0.0000 | | 1.8092 | | 0.9364 | 3.9004 | | 0.6901 | | 926.3941 | | 0.0000 |
| G × B × I(DAT^2^) | 6 | 54.7327 | 0.0000 | | 0.9207 | | 0.9884 | 5.8340 | | 0.4420 | | 3803.0807 | | 0.0000 |
| C × B × DAT | 2 | 39.3622 | 0.0000 | | 2.0840 | | 0.3527 | 0.3583 | | 0.8360 | | 28.9471 | | 0.0000 |
| C × B × I(DAT^2^) | 2 | 58.7399 | 0.0000 | | 1.3903 | | 0.4990 | 0.3754 | | 0.8289 | | 29.9564 | | 0.0000 |
| G × C × B × DAT | 6 | 44.6300 | 0.0000 | | 2.4590 | | 0.8790 | 4.4740 | | 0.6228 | | 809.1543 | | 0.0000 |
| G × C × B × I(DAT^2^) | 6 | 64.3342 | 0.0000 | | 1.6216 | | 0.9510 | 5.3004 | | 0.5059 | | 2481.9762 | | 0.0000 |
| Term | df | ---------- Chl *a ----------* | | | | *----------* Chl *b ----------* | | | | | *----------* Chl *a+b ----------* | | | |
|  |  | statistic | | p.value | | statistic | | | p.value | | statistic | | p.value | |
| (Intercept) | 1 | 7116.9445 | | 0.0000 | | 2257.1012 | | | 0.0000 | | 10002.8356 | | 0.0000 | |
| Genotype (G) | 3 | 4.8309 | | 0.1846 | | 2.3750 | | | 0.4983 | | 5.2830 | | 0.1522 | |
| CRF (C) | 1 | 8.7313 | | 0.0031 | | 0.5201 | | | 0.4708 | | 7.8662 | | 0.0050 | |
| Bioinput (B) | 2 | 0.8332 | | 0.6593 | | 3.4003 | | | 0.1827 | | 1.4683 | | 0.4799 | |
| DAT | 1 | 2.4490 | | 0.1176 | | 203.4966 | | | 0.0000 | | 5.6908 | | 0.0171 | |
| I(DAT^2^) | 1 | 0.7946 | | 0.3727 | | 318.5496 | | | 0.0000 | | 1.9454 | | 0.1631 | |
| G × C | 3 | 26.5372 | | 0.0000 | | 4.6385 | | | 0.2003 | | 25.0469 | | 0.0000 | |
| G × B | 6 | 5.9746 | | 0.4260 | | 9.0269 | | | 0.1721 | | 5.9563 | | 0.4281 | |
| C × B | 2 | 8.3566 | | 0.0153 | | 3.9162 | | | 0.1411 | | 5.7180 | | 0.0573 | |
| G × DAT | 3 | 3.6676 | | 0.2997 | | 15.5071 | | | 0.0014 | | 2.9697 | | 0.3963 | |
| G × I(DAT^2^) | 3 | 7.0189 | | 0.0713 | | 527.1356 | | | 0.0000 | | 7.9346 | | 0.0474 | |
| C × DAT | 1 | 36.8017 | | 0.0000 | | 511.5378 | | | 0.0000 | | 50.9968 | | 0.0000 | |
| C × I(DAT^2^) | 1 | 19.3241 | | 0.0000 | | 1072.0092 | | | 0.0000 | | 23.9968 | | 0.0000 | |
| B × DAT | 2 | 11.8066 | | 0.0027 | | 128.4363 | | | 0.0000 | | 15.5133 | | 0.0004 | |
| B × I(DAT^2^) | 2 | 13.2384 | | 0.0013 | | 721.3182 | | | 0.0000 | | 17.0341 | | 0.0002 | |
| G × C × B | 6 | 28.7931 | | 0.0001 | | 16.8686 | | | 0.0098 | | 19.8119 | | 0.0030 | |
| G × C × DAT | 3 | 77.9130 | | 0.0000 | | 57.5806 | | | 0.0000 | | 69.7686 | | 0.0000 | |
| G × C × I(DAT^2^) | 3 | 66.5731 | | 0.0000 | | 962.8025 | | | 0.0000 | | 55.9186 | | 0.0000 | |
| G × B × DAT | 6 | 12.0938 | | 0.0599 | | 135.9368 | | | 0.0000 | | 14.9803 | | 0.0204 | |
| G × B × I(DAT^2^) | 6 | 15.2154 | | 0.0186 | | 682.3197 | | | 0.0000 | | 19.1385 | | 0.0039 | |
| C × B × DAT | 2 | 5.5454 | | 0.0625 | | 117.6608 | | | 0.0000 | | 9.2323 | | 0.0099 | |
| C × B × I(DAT^2^) | 2 | 6.5272 | | 0.0383 | | 579.8365 | | | 0.0000 | | 10.4645 | | 0.0053 | |
| G × C × B × DAT | 6 | 21.1396 | | 0.0017 | | 282.5488 | | | 0.0000 | | 22.0524 | | 0.0012 | |
| G × C × B × I(DAT^2^) | 6 | 18.3944 | | 0.0053 | | 698.6270 | | | 0.0000 | | 20.6252 | | 0.0021 | |

^a^ Summary of Analysis of Deviance (Type III ANOVA) of longitudinal polynomial models (0-120 DAT). The statistic value refers to the F-test (for SD) or the Wald χ^2^ test (for all other GLMM variables). The terms DAT and I(DAT^2^) represent the linear and quadratic components of time. The high significance (p < 0.05) of high-order interactions involving DAT (e.g., G × C × B × DAT) confirms that growth rates differed among treatments over time and justifies subsequent analyses.

**Table S2.** Pairwise comparisons for growth rates (slopes) of Bioinputs (*Control*, Tríppel^®^, Torpeno^®^) at the experiment midpoint (60 DAT).

| Variable | Genotype | CRF | contrast | estimate | SE | p.adj |
| --- | --- | --- | --- | --- | --- | --- |
| SD | “NR0060408” | 4 g dm^-3^ | *Control* – Tríppel^®^ | -0.0037 | 0.0013 | 0.0162 |
| SD | “NR0060408” | 4 g dm^-3^ | *Control* – Torpeno^®^ | -0.0033 | 0.0013 | 0.0376 |
| SD | “NR0160305” | 4 g dm^-3^ | *Control* – Tríppel^®^ | -0.0037 | 0.0013 | 0.0169 |
| SD | “NR0160305” | 4 g dm^-3^ | *Control* – Torpeno^®^ | -0.0032 | 0.0013 | 0.0408 |
| SD | “Okinawa Roxo” | 4 g dm^-3^ | *Control* – Tríppel^®^ | -0.0092 | 0.0013 | 0.0000 |
| SD | ‘Okinawa Roxo” | 4 g dm^-3^ | *Control* – Torpeno^®^ | -0.0053 | 0.0013 | 0.0002 |
| SD | “Okinawa Roxo” | 4 g dm^-3^ | Tríppel^®^ – Torpeno^®^ | 0.0039 | 0.0013 | 0.0098 |
| SD | ‘Capdeboscq’ | 4 g dm^-3^ | Tríppel^®^ – Torpeno^®^ | 0.0033 | 0.0013 | 0.0333 |
| NL | “NR0060408” | 4 g dm^-3^ | Tríppel^®^ – Torpeno^®^ | -0.0031 | 0.0012 | 0.0325 |
| NL | “Okinawa Roxo” | 4 g dm^-3^ | *Control* – Tríppel^®^ | 0.0033 | 0.0013 | 0.0307 |
| NA | “NR0060408” | 0 g dm^-3^ | *Control* – Tríppel^®^ | -0.0031 | 0.0008 | 0.0008 |
| NA | “NR0160305” | 0 g dm^-3^ | *Control* – Torpeno^®^ | 0.0024 | 0.0009 | 0.0246 |
| NA | “NR0160305” | 0 g dm^-3^ | Tríppel^®^ – Torpeno^®^ | 0.0038 | 0.0010 | 0.0004 |
| NA | “Okinawa Roxo” | 0 g dm^-3^ | *Control* – Torpeno^®^ | -0.0041 | 0.0009 | 0.0000 |
| NA | “Okinawa Roxo” | 0 g dm^-3^ | Tríppel^®^ – Torpeno^®^ | -0.0041 | 0.0010 | 0.0001 |
| NA | “NR0060408” | 4 g dm^-3^ | *Control* – Torpeno^®^ | -0.0025 | 0.0009 | 0.0169 |
| NA | “NR0060408” | 4 g dm^-3^ | Tríppel^®^ – Torpeno^®^ | -0.0043 | 0.0010 | 0.0000 |
| NA | “NR0160305” | 4 g dm^-3^ | Tríppel^®^ – Torpeno^®^ | -0.0026 | 0.0011 | 0.0478 |
| NA | “Okinawa Roxo” | 4 g dm^-3^ | *Control* – Tríppel^®^ | 0.0049 | 0.0010 | 0.0000 |
| NA | “Okinawa Roxo” | 4 g dm^-3^ | *Control* – Torpeno^®^ | 0.0029 | 0.0010 | 0.0081 |
| Chl *a* | “NR0160305” | 0 g dm^-3^ | *Control* – Tríppel^®^ | 0.0019 | 0.0006 | 0.0078 |
| Chl *a* | “NR0160305” | 0 g dm^-3^ | *Control* – Torpeno^®^ | 0.0024 | 0.0006 | 0.0006 |
| Chl *a* | “Okinawa Roxo” | 0 g dm^-3^ | *Control* – Tríppel^®^ | 0.0018 | 0.0006 | 0.0094 |
| Chl *a* | “Okinawa Roxo” | 0 g dm^-3^ | *Control* – Torpeno^®^ | 0.0023 | 0.0006 | 0.0010 |
| Chl *a* | “NR0160305” | 4 g dm^-3^ | *Control* – Torpeno^®^ | 0.0021 | 0.0006 | 0.0023 |
| Chl *a* | “NR0160305” | 4 g dm^-3^ | Tríppel^®^ – Torpeno^®^ | 0.0024 | 0.0006 | 0.0004 |
| Chl *a* | “Okinawa Roxo” | 4 g dm^-3^ | *Control* – Tríppel^®^ | -0.0020 | 0.0006 | 0.0048 |
| Chl *a* | “Okinawa Roxo” | 4 g dm^-3^ | *Control* – Torpeno^®^ | -0.0016 | 0.0006 | 0.0250 |
| Chl *b* | “NR0160305” | 0 g dm^-3^ | *Control* – Tríppel^®^ | 0.0032 | 0.0009 | 0.0012 |
| Chl *b* | “NR0160305” | 0 g dm^-3^ | *Control* – Torpeno^®^ | 0.0023 | 0.0009 | 0.0329 |
| Chl *b* | “NR0160305” | 4 g dm^-3^ | *Control* – Torpeno^®^ | -0.0032 | 0.0010 | 0.0041 |
| Chl *b* | “NR0160305” | 4 g dm^-3^ | Tríppel^®^ – Torpeno^®^ | -0.0048 | 0.0011 | 0.0000 |
| Chl *a+b* | “NR0160305” | 0 g dm^-3^ | *Control* – Tríppel^®^ | 0.0021 | 0.0006 | 0.0006 |
| Chl *a+b* | “NR0160305” | 0 g dm^-3^ | *Control* – Torpeno^®^ | 0.0023 | 0.0006 | 0.0001 |
| Chl *a+b* | “Okinawa Roxo” | 0 g dm^-3^ | *Control* – Tríppel^®^ | 0.0019 | 0.0006 | 0.0027 |
| Chl *a+b* | “Okinawa Roxo” | 0 g dm^-3^ | *Control* – Torpeno^®^ | 0.0021 | 0.0006 | 0.0004 |
| Chl *a+b* | “Okinawa Roxo” | 4 g dm^-3^ | *Control* – Tríppel^®^ | -0.0014 | 0.0006 | 0.0291 |

^a^ Pairwise comparisons of growth rates (slopes) estimated by GLMM models (derived at the midpoint, 60 DAT), evaluating the effect of Bioinputs (*Control*, Tríppel^®^, Torpeno^®^) within each Genotype and CRF scenario. The table displays only statistically significant comparisons (p.adj < 0.05), based on Tukey’s adjustment. *estimate* represents the estimated difference between the growth rates (slope) of the *contrast*. SE = Standard Error. *p.adj* = adjusted p-value.

**Table S3.** Pairwise comparisons for growth rates (slopes) of Genotypes at the experiment midpoint (60 DAT).

| Variable | CRF | Bioinput | contrast | estimate | SE | p.adj |
| --- | --- | --- | --- | --- | --- | --- |
| PH | 4 g dm^-3^ | *Control* | “NR0060408” – “Okinawa Roxo” | 0.0049 | 0.0007 | 0.0000 |
| PH | 4 g dm^-3^ | *Control* | “NR0160305” – “Okinawa Roxo” | 0.0056 | 0.0007 | 0.0000 |
| PH | 4 g dm^-3^ | *Control* | “Okinawa Roxo” – ‘Capdeboscq’ | -0.0064 | 0.0007 | 0.0000 |
| PH | 4 g dm^-3^ | Tríppel^®^ | “NR0060408” – “Okinawa Roxo” | 0.0041 | 0.0007 | 0.0000 |
| PH | 4 g dm^-3^ | Tríppel^®^ | “NR0160305” – “Okinawa Roxo” | 0.0042 | 0.0009 | 0.0000 |
| PH | 4 g dm^-3^ | Tríppel^®^ | “Okinawa Roxo” – ‘Capdeboscq’ | -0.0043 | 0.0009 | 0.0000 |
| PH | 4 g dm^-3^ | Torpeno^®^ | “NR0060408” – “Okinawa Roxo” | 0.0042 | 0.0007 | 0.0000 |
| PH | 4 g dm^-3^ | Torpeno^®^ | “NR0160305” – “Okinawa Roxo” | 0.0042 | 0.0009 | 0.0000 |
| PH | 4 g dm^-3^ | Torpeno^®^ | “Okinawa Roxo” – ‘Capdeboscq’ | -0.0043 | 0.0009 | 0.0000 |
| SD | 4 g dm^-3^ | *Control* | “NR0060408” – “Okinawa Roxo” | 0.0173 | 0.0013 | 0.0000 |
| SD | 4 g dm^-3^ | *Control* | “NR0060408” – ‘Capdeboscq’ | 0.0060 | 0.0013 | 0.0000 |
| SD | 4 g dm^-3^ | *Control* | “NR0160305” – “Okinawa Roxo” | 0.0176 | 0.0013 | 0.0000 |
| SD | 4 g dm^-3^ | *Control* | “NR0160305” – ‘Capdeboscq’ | 0.0063 | 0.0013 | 0.0000 |
| SD | 4 g dm^-3^ | *Control* | “Okinawa Roxo” – ‘Capdeboscq’ | -0.0113 | 0.0013 | 0.0000 |
| SD | 0 g dm^-3^ | Tríppel^®^ | “NR0160305” – ‘Capdeboscq’ | 0.0049 | 0.0013 | 0.0017 |
| SD | 0 g dm^-3^ | Tríppel^®^ | “Okinawa Roxo” – ‘Capdeboscq’ | 0.0045 | 0.0013 | 0.0046 |
| SD | 4 g dm^-3^ | Tríppel^®^ | “NR0060408” – “Okinawa Roxo” | 0.0117 | 0.0013 | 0.0000 |
| SD | 4 g dm^-3^ | Tríppel^®^ | “NR0060408” – ‘Capdeboscq’ | 0.0071 | 0.0013 | 0.0000 |
| SD | 4 g dm^-3^ | Tríppel^®^ | “NR0160305” – “Okinawa Roxo” | 0.0121 | 0.0013 | 0.0000 |
| SD | 4 g dm^-3^ | Tríppel^®^ | “NR0160305” – ‘Capdeboscq’ | 0.0074 | 0.0013 | 0.0000 |
| SD | 4 g dm^-3^ | Tríppel^®^ | “Okinawa Roxo” – ‘Capdeboscq’ | -0.0046 | 0.0013 | 0.0031 |
| SD | 4 g dm^-3^ | Torpeno^®^ | “NR0060408” – “Okinawa Roxo” | 0.0152 | 0.0013 | 0.0000 |
| SD | 4 g dm^-3^ | Torpeno^®^ | “NR0060408” – ‘Capdeboscq’ | 0.0101 | 0.0013 | 0.0000 |
| SD | 4 g dm^-3^ | Torpeno^®^ | “NR0160305” – “Okinawa Roxo” | 0.0155 | 0.0013 | 0.0000 |
| SD | 4 g dm^-3^ | Torpeno^®^ | “NR0160305” – ‘Capdeboscq’ | 0.0103 | 0.0013 | 0.0000 |
| SD | 4 g dm^-3^ | Torpeno^®^ | “Okinawa Roxo” – ‘Capdeboscq’ | -0.0052 | 0.0013 | 0.0006 |
| NL | 0 g dm^-3^ | *Control* | “Okinawa Roxo” – ‘Capdeboscq’ | -0.0044 | 0.0015 | 0.0222 |
| NL | 4 g dm^-3^ | *Control* | “NR0060408” – ‘Capdeboscq’ | -0.0047 | 0.0012 | 0.0008 |
| NL | 4 g dm^-3^ | *Control* | “Okinawa Roxo” – ‘Capdeboscq’ | -0.0053 | 0.0013 | 0.0003 |
| NL | 0 g dm^-3^ | Tríppel^®^ | “NR0160305” – “Okinawa Roxo” | 0.0058 | 0.0015 | 0.0004 |
| NL | 0 g dm^-3^ | Tríppel^®^ | “Okinawa Roxo” – ‘Capdeboscq’ | -0.0042 | 0.0016 | 0.0347 |
| NL | 4 g dm^-3^ | Tríppel^®^ | “NR0060408” – ‘Capdeboscq’ | -0.0062 | 0.0012 | 0.0000 |
| NL | 4 g dm^-3^ | Tríppel^®^ | “NR0160305” – “Okinawa Roxo” | 0.0042 | 0.0012 | 0.0020 |
| NL | 4 g dm^-3^ | Tríppel^®^ | “NR0160305” – ‘Capdeboscq’ | -0.0037 | 0.0011 | 0.0059 |
| NL | 4 g dm^-3^ | Tríppel^®^ | “Okinawa Roxo” – ‘Capdeboscq’ | -0.0079 | 0.0012 | 0.0000 |
| NL | 4 g dm^-3^ | Torpeno^®^ | “NR0060408” – ‘Capdeboscq’ | -0.0034 | 0.0012 | 0.0239 |
| NL | 4 g dm^-3^ | Torpeno^®^ | “NR0160305” – “Okinawa Roxo” | 0.0039 | 0.0012 | 0.0053 |
| NL | 4 g dm^-3^ | Torpeno^®^ | “Okinawa Roxo” – ‘Capdeboscq’ | -0.0061 | 0.0012 | 0.0000 |
| LA | 0 g dm^-3^ | *Control* | “NR0060408” – “NR0160305” | -0.0085 | 0.0008 | 0.0000 |
| LA | 0 g dm^-3^ | *Control* | “NR0060408” – “Okinawa Roxo” | -0.0029 | 0.0008 | 0.0034 |
| LA | 0 g dm^-3^ | *Control* | “NR0060408” – ‘Capdeboscq’ | -0.0094 | 0.0008 | 0.0000 |
| LA | 0 g dm^-3^ | *Control* | “NR0160305” – “Okinawa Roxo” | 0.0055 | 0.0009 | 0.0000 |
| LA | 0 g dm^-3^ | *Control* | “Okinawa Roxo” – ‘Capdeboscq’ | -0.0065 | 0.0009 | 0.0000 |
| LA | 4 g dm^-3^ | *Control* | “NR0060408” – “NR0160305” | -0.0031 | 0.0009 | 0.0037 |
| LA | 4 g dm^-3^ | *Control* | “NR0060408” – ‘Capdeboscq’ | -0.0065 | 0.0009 | 0.0000 |
| LA | 4 g dm^-3^ | *Control* | “NR0160305” – ‘Capdeboscq’ | -0.0034 | 0.0010 | 0.0030 |
| LA | 4 g dm^-3^ | *Control* | “Okinawa Roxo” – ‘Capdeboscq’ | -0.0043 | 0.0010 | 0.0001 |
| LA | 0 g dm^-3^ | Tríppel^®^ | “NR0060408” – “NR0160305” | -0.0068 | 0.0009 | 0.0000 |
| LA | 0 g dm^-3^ | Tríppel^®^ | “NR0060408” – ‘Capdeboscq’ | -0.0057 | 0.0009 | 0.0000 |
| LA | 0 g dm^-3^ | Tríppel^®^ | “NR0160305” – “Okinawa Roxo” | 0.0070 | 0.0010 | 0.0000 |
| LA | 0 g dm^-3^ | Tríppel^®^ | “Okinawa Roxo” – ‘Capdeboscq’ | -0.0059 | 0.0010 | 0.0000 |
| LA | 4 g dm^-3^ | Tríppel^®^ | “NR0060408” – “NR0160305” | -0.0031 | 0.0010 | 0.0072 |
| LA | 4 g dm^-3^ | Tríppel^®^ | “NR0060408” – ‘Capdeboscq’ | -0.0098 | 0.0010 | 0.0000 |
| LA | 4 g dm^-3^ | Tríppel^®^ | “NR0160305” – “Okinawa Roxo” | 0.0040 | 0.0011 | 0.0016 |
| LA | 4 g dm^-3^ | Tríppel^®^ | “NR0160305” – ‘Capdeboscq’ | -0.0066 | 0.0011 | 0.0000 |
| LA | 4 g dm^-3^ | Tríppel^®^ | “Okinawa Roxo” – ‘Capdeboscq’ | -0.0106 | 0.0011 | 0.0000 |
| LA | 0 g dm^-3^ | Torpeno^®^ | “NR0060408” – “NR0160305” | -0.0045 | 0.0009 | 0.0000 |
| LA | 0 g dm^-3^ | Torpeno^®^ | “NR0060408” – “Okinawa Roxo” | -0.0054 | 0.0009 | 0.0000 |
| LA | 0 g dm^-3^ | Torpeno^®^ | “NR0060408” – ‘Capdeboscq’ | -0.0078 | 0.0009 | 0.0000 |
| LA | 0 g dm^-3^ | Torpeno^®^ | “NR0160305” – ‘Capdeboscq’ | -0.0033 | 0.0010 | 0.0050 |
| LA | 4 g dm^-3^ | Torpeno^®^ | “NR0060408” – “Okinawa Roxo” | 0.0032 | 0.0010 | 0.0060 |
| LA | 4 g dm^-3^ | Torpeno^®^ | “NR0060408” – ‘Capdeboscq’ | -0.0044 | 0.0010 | 0.0000 |
| LA | 4 g dm^-3^ | Torpeno^®^ | “NR0160305” – “Okinawa Roxo” | 0.0046 | 0.0011 | 0.0001 |
| LA | 4 g dm^-3^ | Torpeno^®^ | “NR0160305” – ‘Capdeboscq’ | -0.0030 | 0.0011 | 0.0284 |
| LA | 4 g dm^-3^ | Torpeno^®^ | “Okinawa Roxo” – ‘Capdeboscq’ | -0.0076 | 0.0011 | 0.0000 |
| Chl *a* | 0 g dm^-3^ | *Control* | “NR0060408” – “NR0160305” | 0.0028 | 0.0006 | 0.0000 |
| Chl *a* | 0 g dm^-3^ | *Control* | “NR0060408” – ‘Capdeboscq’ | 0.0027 | 0.0006 | 0.0001 |
| Chl *a* | 0 g dm^-3^ | *Control* | “NR0160305” – “Okinawa Roxo” | -0.0023 | 0.0006 | 0.0011 |
| Chl *a* | 0 g dm^-3^ | *Control* | “Okinawa Roxo” – ‘Capdeboscq’ | 0.0022 | 0.0006 | 0.0021 |
| Chl *a* | 4 g dm^-3^ | *Control* | “NR0060408” – ‘Capdeboscq’ | -0.0032 | 0.0006 | 0.0000 |
| Chl *a* | 4 g dm^-3^ | *Control* | “Okinawa Roxo” – ‘Capdeboscq’ | -0.0027 | 0.0006 | 0.0001 |
| Chl *a* | 0 g dm^-3^ | Tríppel^®^ | “NR0060408” – “NR0160305” | 0.0045 | 0.0006 | 0.0000 |
| Chl *a* | 0 g dm^-3^ | Tríppel^®^ | “NR0060408” – “Okinawa Roxo” | 0.0021 | 0.0006 | 0.0050 |
| Chl *a* | 0 g dm^-3^ | Tríppel^®^ | “NR0160305” – “Okinawa Roxo” | -0.0024 | 0.0006 | 0.0009 |
| Chl *a* | 0 g dm^-3^ | Tríppel^®^ | “NR0160305” – “Capdeboscq’ | -0.0031 | 0.0006 | 0.0000 |
| Chl *a* | 0 g dm^-3^ | Torpeno^®^ | “NR0060408” – “NR0160305” | 0.0050 | 0.0006 | 0.0000 |
| Chl *a* | 0 g dm^-3^ | Torpeno^®^ | “NR0060408” – “Okinawa Roxo” | 0.0025 | 0.0006 | 0.0003 |
| Chl *a* | 0 g dm^-3^ | Torpeno^®^ | “NR0060408” – ‘Capdeboscq’ | 0.0017 | 0.0006 | 0.0349 |
| Chl *a* | 0 g dm^-3^ | Torpeno^®^ | “NR0160305” – “Okinawa Roxo” | -0.0024 | 0.0006 | 0.0006 |
| Chl *a* | 0 g dm^-3^ | Torpeno^®^ | “NR0160305” – ‘Capdeboscq’ | -0.0033 | 0.0006 | 0.0000 |
| Chl *a* | 4 g dm^-3^ | Torpeno^®^ | “NR0060408” – “NR0160305” | 0.0019 | 0.0006 | 0.0106 |
| Chl *a* | 4 g dm^-3^ | Torpeno^®^ | “NR0060408” – ‘Capdeboscq’ | -0.0017 | 0.0006 | 0.0289 |
| Chl *a* | 4 g dm^-3^ | Torpeno^®^ | “NR0160305” – “Okinawa Roxo” | -0.0026 | 0.0006 | 0.0002 |
| Chl *a* | 4 g dm^-3^ | Torpeno^®^ | “NR0160305” – ‘Capdeboscq’ | -0.0037 | 0.0006 | 0.0000 |
| Chl *b* | 0 g dm^-3^ | *Control* | “NR0060408” – ‘Capdeboscq’ | 0.0048 | 0.0008 | 0.0000 |
| Chl *b* | 0 g dm^-3^ | *Control* | “NR0160305” – ‘Capdeboscq’ | 0.0028 | 0.0009 | 0.0089 |
| Chl *b* | 0 g dm^-3^ | *Control* | “Okinawa Roxo” – ‘Capdeboscq’ | 0.0037 | 0.0009 | 0.0002 |
| Chl *b* | 4 g dm^-3^ | *Control* | “NR0060408” – “Okinawa Roxo” | 0.0027 | 0.0009 | 0.0100 |
| Chl *b* | 4 g dm^-3^ | *Control* | “NR0060408” – ‘Capdeboscq’ | -0.0033 | 0.0009 | 0.0011 |
| Chl *b* | 4 g dm^-3^ | *Control* | “NR0160305” – ‘Capdeboscq’ | -0.0036 | 0.0010 | 0.0010 |
| Chl *b* | 4 g dm^-3^ | *Control* | “Okinawa Roxo” – ‘Capdeboscq’ | -0.006 | 0.0010 | 0.0000 |
| Chl *b* | 0 g dm^-3^ | Tríppel^®^ | “NR0060408” – “NR0160305” | 0.0052 | 0.0009 | 0.0000 |
| Chl *b* | 0 g dm^-3^ | Tríppel^®^ | “NR0060408” – “Okinawa Roxo” | 0.0030 | 0.0009 | 0.0055 |
| Chl *b* | 0 g dm^-3^ | Tríppel^®^ | “NR0060408” – ‘Capdeboscq’ | 0.0048 | 0.0009 | 0.0000 |
| Chl *b* | 4 g dm^-3^ | Tríppel^®^ | “NR0060408” – “Okinawa Roxo” | 0.0045 | 0.0010 | 0.0001 |
| Chl *b* | 4 g dm^-3^ | Tríppel^®^ | “NR0160305” – ‘Capdeboscq’ | -0.0049 | 0.0011 | 0.0000 |
| Chl *b* | 4 g dm^-3^ | Tríppel^®^ | “Okinawa Roxo” – ‘Capdeboscq’ | -0.0069 | 0.0011 | 0.0000 |
| Chl *b* | 0 g dm^-3^ | Torpeno^®^ | “NR0060408” – “NR0160305” | 0.0038 | 0.0009 | 0.0002 |
| Chl *b* | 0 g dm^-3^ | Torpeno^®^ | “NR0060408” – ‘Capdeboscq’ | 0.0040 | 0.0009 | 0.0001 |
| Chl *b* | 4 g dm^-3^ | Torpeno^®^ | “NR0060408” – “Okinawa Roxo” | 0.0047 | 0.0010 | 0.0000 |
| Chl *b* | 4 g dm^-3^ | Torpeno^®^ | “NR0160305” – “Okinawa Roxo” | 0.0069 | 0.0011 | 0.0000 |
| Chl *b* | 4 g dm^-3^ | Torpeno^®^ | “Okinawa Roxo” – ‘Capdeboscq’ | -0.0064 | 0.0011 | 0.0000 |
| Chl *a+b* | 0 g dm^-3^ | *Control* | “NR0060408” – “NR0160305” | 0.0027 | 0.0006 | 0.0000 |
| Chl *a+b* | 0 g dm^-3^ | *Control* | “NR0060408” – ‘Capdeboscq’ | 0.0031 | 0.0006 | 0.0000 |
| Chl *a+b* | 0 g dm^-3^ | *Control* | “NR0160305” – “Okinawa Roxo” | -0.0021 | 0.0006 | 0.0013 |
| Chl *a+b* | 0 g dm^-3^ | *Control* | “Okinawa Roxo” – ‘Capdeboscq’ | 0.0025 | 0.0006 | 0.0001 |
| Chl *a+b* | 4 g dm^-3^ | *Control* | “NR0060408” – ‘Capdeboscq’ | -0.0032 | 0.0006 | 0.0000 |
| Chl *a+b* | 4 g dm^-3^ | *Control* | “NR0160305” – ‘Capdeboscq’ | -0.0020 | 0.0006 | 0.0017 |
| Chl *a+b* | 4 g dm^-3^ | *Control* | “Okinawa Roxo” – ‘Capdeboscq’ | -0.0034 | 0.0006 | 0.0000 |
| Chl *a+b* | 0 g dm^-3^ | Tríppel^®^ | “NR0060408” – “NR0160305” | 0.0046 | 0.0006 | 0.0000 |
| Chl *a+b* | 0 g dm^-3^ | Tríppel^®^ | “NR0060408” – “Okinawa Roxo” | 0.0023 | 0.0006 | 0.0003 |
| Chl *a+b* | 0 g dm^-3^ | Tríppel^®^ | “NR0060408” – ‘Capdeboscq’ | 0.0020 | 0.0006 | 0.0019 |
| Chl *a+b* | 0 g dm^-3^ | Tríppel^®^ | “NR0160305” – “Okinawa Roxo” | -0.0023 | 0.0006 | 0.0002 |
| Chl *a+b* | 0 g dm^-3^ | Tríppel^®^ | “NR0160305” – ‘Capdeboscq’ | -0.0026 | 0.0006 | 0.0000 |
| Chl *a+b* | 0 g dm^-3^ | Torpeno^®^ | “NR0060408” – “NR0160305” | 0.0047 | 0.0006 | 0.0000 |
| Chl *a+b* | 0 g dm^-3^ | Torpeno^®^ | “NR0060408” – “Okinawa Roxo” | 0.0025 | 0.0006 | 0.0001 |
| Chl *a+b* | 0 g dm^-3^ | Torpeno^®^ | “NR0060408” – ‘Capdeboscq’ | 0.0020 | 0.0006 | 0.0016 |
| Chl *a+b* | 0 g dm^-3^ | Torpeno^®^ | “NR0160305” – “Okinawa Roxo” | -0.0023 | 0.0006 | 0.0003 |
| Chl *a+b* | 0 g dm^-3^ | Torpeno^®^ | “NR0160305” – ‘Capdeboscq’ | -0.0027 | 0.0006 | 0.0000 |
| Chl *a+b* | 4 g dm^-3^ | Torpeno^®^ | “NR0060408” – ‘Capdeboscq’ | -0.0017 | 0.0006 | 0.0118 |
| Chl *a+b* | 4 g dm^-3^ | Torpeno^®^ | “NR0160305” – ‘Capdeboscq’ | -0.0029 | 0.0006 | 0.0000 |
| Chl *a+b* | 4 g dm^-3^ | Torpeno^®^ | “Okinawa Roxo” – ‘Capdeboscq’ | -0.0021 | 0.0006 | 0.0008 |

^a^ Pairwise comparisons of growth rates (slopes) estimated by GLMM models (derived at the midpoint, 60 DAT), evaluating the effect of Genotypes within each CRF and Bioinput scenario. The table displays only statistically significant comparisons (p.adj < 0.05), based on Tukey’s adjustment. *estimate* represents the estimated difference between the growth rates (slope) of the *contrast*. SE = Standard Error. *p.adj* = adjusted p-value.

**Table S4.** Means of growth variables (PH, SD, NL, LA) and chlorophyll (Chl *a*, *b*, *a+b*) at seven evaluation points (0-120 DAT) for four rootstock genotypes under different CRF and Bioinput treatments.

| Genotype | CRF | Bioinput | DAT | PH | SD | NL | LA | Chl *a* | Chl *b* | Chl *a+b* |
| --- | --- | --- | --- | --- | --- | --- | --- | --- | --- | --- |
| ‘Capdeboscq’ | 0 g dm^-3^ | *Control* | 0 | 22.40 | 1.72 | 17.8 | 177.12 | 32.82 | 9.04 | 41.86 |
| ‘Capdeboscq’ | 0 g dm^-3^ | *Control* | 20 | 36.42 | 2.08 | 19.6 | 357.66 | 35.60 | 9.72 | 45.32 |
| ‘Capdeboscq’ | 0 g dm^-3^ | *Control* | 40 | 46.16 | 2.42 | 22.6 | 497.38 | 32.02 | 8.60 | 40.62 |
| ‘Capdeboscq’ | 0 g dm^-3^ | *Control* | 60 | 46.40 | 2.69 | 24.4 | 532.57 | 30.48 | 6.58 | 37.06 |
| ‘Capdeboscq’ | 0 g dm^-3^ | *Control* | 80 | 46.70 | 2.83 | 23.2 | 489.16 | 24.76 | 5.26 | 30.02 |
| ‘Capdeboscq’ | 0 g dm^-3^ | *Control* | 100 | 47.36 | 2.89 | 15.2 | 305.14 | 23.56 | 4.40 | 27.96 |
| ‘Capdeboscq’ | 0 g dm^-3^ | *Control* | 120 | 47.76 | 2.97 | 11.2 | 210.67 | 23.06 | 4.28 | 27.34 |
| ‘Capdeboscq’ | 4 g dm^-3^ | *Control* | 0 | 22.80 | 1.51 | 14.2 | 125.06 | 28.22 | 8.62 | 36.84 |
| ‘Capdeboscq’ | 4 g dm^-3^ | *Control* | 20 | 33.70 | 1.88 | 17.8 | 357.01 | 37.42 | 10.40 | 47.82 |
| ‘Capdeboscq’ | 4 g dm^-3^ | *Control* | 40 | 50.90 | 2.39 | 24.4 | 631.75 | 52.02 | 12.18 | 64.20 |
| ‘Capdeboscq’ | 4 g dm^-3^ | *Control* | 60 | 68.62 | 3.29 | 35.0 | 964.37 | 51.72 | 13.08 | 64.80 |
| ‘Capdeboscq’ | 4 g dm^-3^ | *Control* | 80 | 81.46 | 3.96 | 41.0 | 1458.00 | 64.20 | 15.24 | 79.44 |
| ‘Capdeboscq’ | 4 g dm^-3^ | *Control* | 100 | 87.70 | 4.30 | 45.2 | 1478.87 | 57.98 | 21.82 | 79.80 |
| ‘Capdeboscq’ | 4 g dm^-3^ | *Control* | 120 | 91.96 | 4.76 | 43.2 | 1433.61 | 62.56 | 21.60 | 84.16 |
| ‘Capdeboscq’ | 0 g dm^-3^ | Tríppel^®^ | 0 | 21.56 | 1.64 | 16.0 | 143.20 | 29.20 | 9.04 | 38.24 |
| ‘Capdeboscq’ | 0 g dm^-3^ | Tríppel^®^ | 20 | 35.34 | 2.01 | 18.0 | 357.11 | 33.36 | 8.94 | 42.30 |
| ‘Capdeboscq’ | 0 g dm^-3^ | Tríppel^®^ | 40 | 44.78 | 2.16 | 21.4 | 444.01 | 29.34 | 7.92 | 37.26 |
| ‘Capdeboscq’ | 0 g dm^-3^ | Tríppel^®^ | 60 | 45.06 | 2.48 | 22.4 | 438.14 | 27.70 | 6.10 | 33.80 |
| ‘Capdeboscq’ | 0 g dm^-3^ | Tríppel^®^ | 80 | 45.36 | 2.61 | 23.6 | 514.26 | 24.96 | 4.66 | 29.62 |
| ‘Capdeboscq’ | 0 g dm^-3^ | Tríppel^®^ | 100 | 46.42 | 2.73 | 14.6 | 247.45 | 23.90 | 4.36 | 28.26 |
| ‘Capdeboscq’ | 0 g dm^-3^ | Tríppel^®^ | 120 | 46.60 | 2.75 | 9.4 | 165.53 | 23.18 | 4.12 | 27.30 |
| ‘Capdeboscq’ | 4 g dm^-3^ | Tríppel^®^ | 0 | 25.30 | 1.63 | 17.6 | 158.60 | 34.16 | 9.86 | 44.02 |
| ‘Capdeboscq’ | 4 g dm^-3^ | Tríppel^®^ | 20 | 38.34 | 2.14 | 21.2 | 336.38 | 36.24 | 12.14 | 48.38 |
| ‘Capdeboscq’ | 4 g dm^-3^ | Tríppel^®^ | 40 | 55.70 | 2.60 | 27.2 | 751.17 | 51.78 | 11.92 | 63.70 |
| ‘Capdeboscq’ | 4 g dm^-3^ | Tríppel^®^ | 60 | 73.80 | 3.32 | 41.6 | 1250.22 | 61.58 | 13.76 | 75.34 |
| ‘Capdeboscq’ | 4 g dm^-3^ | Tríppel^®^ | 80 | 90.26 | 4.14 | 43.6 | 1504.89 | 56.12 | 19.56 | 75.68 |
| ‘Capdeboscq’ | 4 g dm^-3^ | Tríppel^®^ | 100 | 99.80 | 4.81 | 49.2 | 1929.84 | 61.12 | 23.48 | 84.60 |
| ‘Capdeboscq’ | 4 g dm^-3^ | Tríppel^®^ | 120 | 99.80 | 5.20 | 51.2 | 1990.06 | 59.98 | 22.62 | 82.60 |
| ‘Capdeboscq’ | 0 g dm^-3^ | Torpeno^®^ | 0 | 23.20 | 1.54 | 14.4 | 111.61 | 33.06 | 9.24 | 42.30 |
| ‘Capdeboscq’ | 0 g dm^-3^ | Torpeno^®^ | 20 | 38.62 | 2.00 | 18.8 | 374.86 | 36.84 | 8.24 | 45.08 |
| ‘Capdeboscq’ | 0 g dm^-3^ | Torpeno^®^ | 40 | 47.72 | 2.21 | 21.8 | 457.94 | 31.64 | 8.04 | 39.68 |
| ‘Capdeboscq’ | 0 g dm^-3^ | Torpeno^®^ | 60 | 48.60 | 2.48 | 25.6 | 520.59 | 29.38 | 6.56 | 35.94 |
| ‘Capdeboscq’ | 0 g dm^-3^ | Torpeno^®^ | 80 | 48.60 | 2.66 | 22.0 | 425.33 | 26.40 | 5.22 | 31.62 |
| ‘Capdeboscq’ | 0 g dm^-3^ | Torpeno^®^ | 100 | 49.70 | 2.71 | 16.0 | 310.02 | 24.08 | 4.28 | 28.36 |
| ‘Capdeboscq’ | 0 g dm^-3^ | Torpeno^®^ | 120 | 50.12 | 2.76 | 9.2 | 143.95 | 26.54 | 4.10 | 30.64 |
| ‘Capdeboscq’ | 4 g dm^-3^ | Torpeno^®^ | 0 | 23.06 | 1.75 | 17.6 | 150.13 | 34.02 | 10.20 | 44.22 |
| ‘Capdeboscq’ | 4 g dm^-3^ | Torpeno^®^ | 20 | 38.30 | 2.16 | 18.6 | 435.74 | 36.32 | 10.88 | 47.20 |
| ‘Capdeboscq’ | 4 g dm^-3^ | Torpeno^®^ | 40 | 50.90 | 2.56 | 24.6 | 676.87 | 37.68 | 11.68 | 49.36 |
| ‘Capdeboscq’ | 4 g dm^-3^ | Torpeno^®^ | 60 | 68.04 | 3.23 | 36.0 | 1052.03 | 37.48 | 12.44 | 49.92 |
| ‘Capdeboscq’ | 4 g dm^-3^ | Torpeno^®^ | 80 | 81.24 | 3.89 | 43.2 | 1456.90 | 55.06 | 17.80 | 72.86 |
| ‘Capdeboscq’ | 4 g dm^-3^ | Torpeno^®^ | 100 | 93.26 | 4.41 | 45.2 | 1730.48 | 62.32 | 20.60 | 82.92 |
| ‘Capdeboscq’ | 4 g dm^-3^ | Torpeno^®^ | 120 | 95.34 | 5.05 | 50.8 | 1913.85 | 66.26 | 21.86 | 88.12 |
| “Okinawa Roxo” | 0 g dm^-3^ | *Control* | 0 | 29.78 | 1.75 | 20.0 | 239.53 | 29.70 | 8.82 | 38.52 |
| “Okinawa Roxo” | 0 g dm^-3^ | *Control* | 20 | 52.26 | 2.48 | 25.8 | 463.84 | 34.30 | 8.16 | 42.46 |
| “Okinawa Roxo” | 0 g dm^-3^ | *Control* | 40 | 62.46 | 2.75 | 29.8 | 589.92 | 30.04 | 6.94 | 36.98 |
| “Okinawa Roxo” | 0 g dm^-3^ | *Control* | 60 | 64.56 | 2.98 | 33.4 | 588.11 | 29.30 | 6.16 | 35.46 |
| “Okinawa Roxo” | 0 g dm^-3^ | *Control* | 80 | 65.90 | 3.32 | 20.2 | 466.50 | 27.96 | 5.60 | 33.56 |
| “Okinawa Roxo” | 0 g dm^-3^ | *Control* | 100 | 66.20 | 3.32 | 12.0 | 220.29 | 30.76 | 6.28 | 37.04 |
| “Okinawa Roxo” | 0 g dm^-3^ | *Control* | 120 | 66.40 | 3.51 | 9.8 | 134.31 | 24.26 | 5.24 | 29.50 |
| “Okinawa Roxo” | 4 g dm^-3^ | *Control* | 0 | 20.30 | 1.38 | 13.4 | 78.90 | 31.84 | 8.92 | 40.76 |
| “Okinawa Roxo” | 4 g dm^-3^ | *Control* | 20 | 32.20 | 1.75 | 17.2 | 214.54 | 37.72 | 9.86 | 47.58 |
| “Okinawa Roxo” | 4 g dm^-3^ | *Control* | 40 | 41.82 | 2.02 | 26.4 | 531.45 | 34.22 | 10.06 | 44.28 |
| “Okinawa Roxo” | 4 g dm^-3^ | *Control* | 60 | 44.96 | 2.67 | 31.2 | 699.75 | 32.98 | 10.38 | 43.36 |
| “Okinawa Roxo” | 4 g dm^-3^ | *Control* | 80 | 47.36 | 3.02 | 34.0 | 861.42 | 34.48 | 9.64 | 44.12 |
| “Okinawa Roxo” | 4 g dm^-3^ | *Control* | 100 | 49.70 | 3.21 | 26.4 | 648.21 | 37.48 | 11.96 | 49.44 |
| “Okinawa Roxo” | 4 g dm^-3^ | *Control* | 120 | 49.80 | 3.36 | 23.6 | 553.01 | 61.08 | 11.54 | 72.62 |
| “Okinawa Roxo” | 0 g dm^-3^ | Tríppel^®^ | 0 | 29.50 | 1.84 | 19.8 | 221.52 | 30.46 | 8.68 | 39.14 |
| “Okinawa Roxo” | 0 g dm^-3^ | Tríppel^®^ | 20 | 52.06 | 2.44 | 26.0 | 564.14 | 32.68 | 8.50 | 41.18 |
| “Okinawa Roxo” | 0 g dm^-3^ | Tríppel^®^ | 40 | 64.00 | 2.77 | 32.8 | 682.83 | 31.40 | 7.82 | 39.22 |
| “Okinawa Roxo” | 0 g dm^-3^ | Tríppel^®^ | 60 | 64.90 | 3.20 | 32.6 | 571.03 | 28.64 | 6.92 | 35.56 |
| “Okinawa Roxo” | 0 g dm^-3^ | Tríppel^®^ | 80 | 66.10 | 3.38 | 21.2 | 482.93 | 25.30 | 5.44 | 30.74 |
| “Okinawa Roxo” | 0 g dm^-3^ | Tríppel^®^ | 100 | 66.50 | 3.43 | 12.8 | 240.85 | 24.52 | 5.46 | 29.98 |
| “Okinawa Roxo” | 0 g dm^-3^ | Tríppel^®^ | 120 | 66.50 | 3.56 | 10.2 | 140.70 | 20.92 | 4.32 | 25.24 |
| “Okinawa Roxo” | 4 g dm^-3^ | Tríppel^®^ | 0 | 31.40 | 1.65 | 20.6 | 192.37 | 31.62 | 9.12 | 40.74 |
| “Okinawa Roxo” | 4 g dm^-3^ | Tríppel^®^ | 20 | 48.60 | 2.03 | 24.0 | 448.20 | 37.08 | 9.38 | 46.46 |
| “Okinawa Roxo” | 4 g dm^-3^ | Tríppel^®^ | 40 | 62.24 | 2.56 | 32.6 | 730.72 | 37.92 | 10.34 | 48.26 |
| “Okinawa Roxo” | 4 g dm^-3^ | Tríppel^®^ | 60 | 70.20 | 3.37 | 38.0 | 1128.63 | 36.26 | 11.14 | 47.40 |
| “Okinawa Roxo” | 4 g dm^-3^ | Tríppel^®^ | 80 | 77.30 | 4.02 | 42.4 | 1228.93 | 35.34 | 9.54 | 44.88 |
| “Okinawa Roxo” | 4 g dm^-3^ | Tríppel^®^ | 100 | 82.60 | 4.32 | 28.8 | 882.85 | 59.08 | 10.44 | 69.52 |
| “Okinawa Roxo” | 4 g dm^-3^ | Tríppel^®^ | 120 | 83.00 | 4.64 | 22.2 | 720.97 | 65.28 | 10.14 | 75.42 |
| “Okinawa Roxo” | 0 g dm^-3^ | Torpeno^®^ | 0 | 28.24 | 1.64 | 19.6 | 201.73 | 30.56 | 7.76 | 38.32 |
| “Okinawa Roxo” | 0 g dm^-3^ | Torpeno^®^ | 20 | 49.30 | 2.22 | 23.8 | 377.20 | 33.88 | 7.76 | 41.64 |
| “Okinawa Roxo” | 0 g dm^-3^ | Torpeno^®^ | 40 | 59.78 | 2.52 | 30.2 | 685.16 | 30.06 | 7.20 | 37.26 |
| “Okinawa Roxo” | 0 g dm^-3^ | Torpeno^®^ | 60 | 60.00 | 2.94 | 32.8 | 653.84 | 29.30 | 5.64 | 34.94 |
| “Okinawa Roxo” | 0 g dm^-3^ | Torpeno^®^ | 80 | 61.30 | 3.14 | 21.4 | 492.24 | 26.64 | 5.78 | 32.42 |
| “Okinawa Roxo” | 0 g dm^-3^ | Torpeno^®^ | 100 | 62.10 | 3.20 | 16.2 | 316.41 | 25.14 | 4.94 | 30.08 |
| “Okinawa Roxo” | 0 g dm^-3^ | Torpeno^®^ | 120 | 62.20 | 3.20 | 12.4 | 173.20 | 18.94 | 3.84 | 22.78 |
| “Okinawa Roxo” | 4 g dm^-3^ | Torpeno^®^ | 0 | 26.00 | 1.56 | 18.0 | 143.54 | 32.60 | 9.54 | 42.14 |
| “Okinawa Roxo” | 4 g dm^-3^ | Torpeno^®^ | 20 | 40.90 | 2.00 | 22.8 | 430.91 | 36.10 | 9.52 | 45.62 |
| “Okinawa Roxo” | 4 g dm^-3^ | Torpeno^®^ | 40 | 56.22 | 2.31 | 29.6 | 636.71 | 34.86 | 10.20 | 45.06 |
| “Okinawa Roxo” | 4 g dm^-3^ | Torpeno^®^ | 60 | 62.10 | 3.04 | 34.4 | 726.43 | 37.62 | 9.62 | 47.24 |
| “Okinawa Roxo” | 4 g dm^-3^ | Torpeno^®^ | 80 | 67.50 | 3.56 | 38.4 | 1046.66 | 34.14 | 8.80 | 42.94 |
| “Okinawa Roxo” | 4 g dm^-3^ | Torpeno^®^ | 100 | 70.50 | 4.01 | 32.4 | 975.11 | 59.30 | 11.12 | 70.42 |
| “Okinawa Roxo” | 4 g dm^-3^ | Torpeno^®^ | 120 | 70.80 | 4.07 | 26.2 | 731.24 | 60.28 | 10.28 | 70.56 |
| “NR0060408” | 0 g dm^-3^ | *Control* | 0 | 28.40 | 2.22 | 20.0 | 271.98 | 33.28 | 8.48 | 41.76 |
| “NR0060408” | 0 g dm^-3^ | *Control* | 20 | 50.64 | 2.49 | 24.4 | 549.43 | 34.10 | 9.48 | 43.58 |
| “NR0060408” | 0 g dm^-3^ | *Control* | 40 | 62.56 | 2.76 | 28.4 | 635.92 | 29.92 | 7.22 | 37.14 |
| “NR0060408” | 0 g dm^-3^ | *Control* | 60 | 63.80 | 3.29 | 26.6 | 542.03 | 30.24 | 6.82 | 37.06 |
| “NR0060408” | 0 g dm^-3^ | *Control* | 80 | 64.10 | 3.42 | 21.4 | 454.52 | 28.98 | 5.70 | 34.68 |
| “NR0060408” | 0 g dm^-3^ | *Control* | 100 | 64.90 | 3.48 | 14.6 | 227.04 | 31.42 | 6.96 | 38.38 |
| “NR0060408” | 0 g dm^-3^ | *Control* | 120 | 64.90 | 3.52 | 9.0 | 102.24 | 28.76 | 6.50 | 35.26 |
| “NR0060408” | 4 g dm^-3^ | *Control* | 0 | 26.90 | 1.89 | 18.6 | 220.75 | 33.48 | 9.58 | 43.06 |
| “NR0060408” | 4 g dm^-3^ | *Control* | 20 | 50.86 | 2.56 | 21.6 | 609.52 | 63.04 | 11.60 | 74.64 |
| “NR0060408” | 4 g dm^-3^ | *Control* | 40 | 72.14 | 3.57 | 28.8 | 895.10 | 56.08 | 15.00 | 71.08 |
| “NR0060408” | 4 g dm^-3^ | *Control* | 60 | 89.50 | 4.12 | 35.8 | 1185.88 | 59.16 | 14.18 | 73.34 |
| “NR0060408” | 4 g dm^-3^ | *Control* | 80 | 101.00 | 4.93 | 41.6 | 1488.37 | 58.02 | 16.40 | 74.42 |
| “NR0060408” | 4 g dm^-3^ | *Control* | 100 | 110.00 | 5.67 | 39.2 | 1601.22 | 60.40 | 17.58 | 77.98 |
| “NR0060408” | 4 g dm^-3^ | *Control* | 120 | 110.20 | 5.87 | 30.8 | 1152.89 | 62.24 | 17.78 | 80.02 |
| “NR0060408” | 0 g dm^-3^ | Tríppel^®^ | 0 | 28.00 | 2.01 | 17.6 | 203.90 | 34.70 | 7.68 | 42.38 |
| “NR0060408” | 0 g dm^-3^ | Tríppel^®^ | 20 | 49.10 | 2.51 | 19.8 | 373.50 | 35.18 | 10.60 | 45.78 |
| “NR0060408” | 0 g dm^-3^ | Tríppel^®^ | 40 | 60.66 | 2.83 | 26.0 | 604.18 | 32.66 | 8.80 | 41.46 |
| “NR0060408” | 0 g dm^-3^ | Tríppel^®^ | 60 | 62.40 | 3.24 | 27.0 | 558.88 | 51.04 | 8.02 | 59.06 |
| “NR0060408” | 0 g dm^-3^ | Tríppel^®^ | 80 | 62.50 | 3.30 | 23.6 | 519.02 | 29.16 | 6.00 | 35.16 |
| “NR0060408” | 0 g dm^-3^ | Tríppel^®^ | 100 | 62.80 | 3.50 | 15.0 | 274.11 | 28.70 | 7.76 | 36.46 |
| “NR0060408” | 0 g dm^-3^ | Tríppel^®^ | 120 | 62.80 | 3.52 | 7.6 | 87.86 | 31.74 | 6.16 | 37.90 |
| “NR0060408” | 4 g dm^-3^ | Tríppel^®^ | 0 | 31.40 | 1.88 | 19.0 | 216.71 | 34.86 | 10.54 | 45.40 |
| “NR0060408” | 4 g dm^-3^ | Tríppel^®^ | 20 | 54.00 | 2.86 | 22.6 | 663.35 | 41.62 | 11.96 | 53.58 |
| “NR0060408” | 4 g dm^-3^ | Tríppel^®^ | 40 | 79.16 | 3.67 | 30.4 | 1097.08 | 52.54 | 19.90 | 72.44 |
| “NR0060408” | 4 g dm^-3^ | Tríppel^®^ | 60 | 103.30 | 4.71 | 40.6 | 1518.62 | 61.76 | 14.28 | 76.04 |
| “NR0060408” | 4 g dm^-3^ | Tríppel^®^ | 80 | 119.60 | 5.46 | 47.8 | 1881.33 | 60.06 | 16.18 | 76.24 |
| “NR0060408” | 4 g dm^-3^ | Tríppel^®^ | 100 | 128.40 | 6.14 | 28.4 | 1157.45 | 57.54 | 21.82 | 79.36 |
| “NR0060408” | 4 g dm^-3^ | Tríppel^®^ | 120 | 128.40 | 6.30 | 25.0 | 1017.51 | 59.42 | 20.82 | 80.24 |
| “NR0060408” | 0 g dm^-3^ | Torpeno^®^ | 0 | 30.40 | 1.81 | 19.4 | 242.86 | 34.80 | 9.30 | 44.10 |
| “NR0060408” | 0 g dm^-3^ | Torpeno^®^ | 20 | 50.64 | 2.54 | 21.0 | 416.28 | 36.22 | 9.52 | 45.74 |
| “NR0060408” | 0 g dm^-3^ | Torpeno^®^ | 40 | 60.86 | 2.76 | 24.0 | 464.55 | 31.14 | 8.48 | 39.62 |
| “NR0060408” | 0 g dm^-3^ | Torpeno^®^ | 60 | 60.96 | 2.95 | 29.8 | 567.25 | 31.80 | 7.38 | 39.18 |
| “NR0060408” | 0 g dm^-3^ | Torpeno^®^ | 80 | 61.30 | 3.32 | 23.0 | 485.13 | 31.20 | 6.16 | 37.36 |
| “NR0060408” | 0 g dm^-3^ | Torpeno^®^ | 100 | 62.20 | 3.44 | 14.8 | 283.61 | 30.90 | 7.08 | 37.98 |
| “NR0060408” | 0 g dm^-3^ | Torpeno^®^ | 120 | 62.20 | 3.49 | 7.6 | 77.06 | 30.24 | 6.54 | 36.78 |
| “NR0060408” | 4 g dm^-3^ | Torpeno^®^ | 0 | 29.64 | 2.09 | 17.8 | 207.36 | 34.64 | 10.04 | 44.68 |
| “NR0060408” | 4 g dm^-3^ | Torpeno^®^ | 20 | 51.34 | 3.04 | 20.8 | 583.72 | 37.82 | 13.40 | 51.22 |
| “NR0060408” | 4 g dm^-3^ | Torpeno^®^ | 40 | 78.28 | 3.77 | 29.4 | 807.87 | 56.18 | 21.02 | 77.20 |
| “NR0060408” | 4 g dm^-3^ | Torpeno^®^ | 60 | 103.80 | 4.48 | 40.8 | 1542.73 | 61.62 | 17.36 | 78.98 |
| “NR0060408” | 4 g dm^-3^ | Torpeno^®^ | 80 | 120.60 | 5.57 | 48.8 | 2183.35 | 58.88 | 18.68 | 77.56 |
| “NR0060408” | 4 g dm^-3^ | Torpeno^®^ | 100 | 126.40 | 6.23 | 41.6 | 1919.31 | 57.26 | 21.84 | 79.10 |
| “NR0060408” | 4 g dm^-3^ | Torpeno^®^ | 120 | 127.20 | 6.48 | 29.2 | 1244.66 | 59.86 | 21.14 | 81.00 |
| “NR0160305” | 0 g dm^-3^ | *Control* | 0 | 25.80 | 2.04 | 20.0 | 130.03 | 32.06 | 8.60 | 40.66 |
| “NR0160305” | 0 g dm^-3^ | *Control* | 20 | 45.22 | 2.68 | 23.2 | 404.60 | 36.70 | 9.94 | 46.64 |
| “NR0160305” | 0 g dm^-3^ | *Control* | 40 | 57.72 | 2.77 | 28.0 | 556.37 | 31.66 | 7.82 | 39.48 |
| “NR0160305” | 0 g dm^-3^ | *Control* | 60 | 58.30 | 3.08 | 34.0 | 597.13 | 29.54 | 6.62 | 36.16 |
| “NR0160305” | 0 g dm^-3^ | *Control* | 80 | 58.90 | 3.36 | 30.8 | 617.26 | 29.40 | 5.82 | 35.22 |
| “NR0160305” | 0 g dm^-3^ | *Control* | 100 | 59.60 | 3.42 | 15.0 | 257.81 | 21.54 | 6.10 | 27.64 |
| “NR0160305” | 0 g dm^-3^ | *Control* | 120 | 59.70 | 3.48 | 11.2 | 152.47 | 22.42 | 5.24 | 27.66 |
| “NR0160305” | 4 g dm^-3^ | *Control* | 0 | 29.00 | 1.97 | 18.6 | 188.92 | 34.04 | 8.88 | 42.92 |
| “NR0160305” | 4 g dm^-3^ | *Control* | 20 | 52.32 | 3.14 | 21.6 | 505.31 | 39.28 | 12.12 | 51.40 |
| “NR0160305” | 4 g dm^-3^ | *Control* | 40 | 79.26 | 3.82 | 36.2 | 955.96 | 51.74 | 17.84 | 69.58 |
| “NR0160305” | 4 g dm^-3^ | *Control* | 60 | 98.00 | 4.75 | 46.8 | 1488.71 | 61.64 | 15.24 | 76.88 |
| “NR0160305” | 4 g dm^-3^ | *Control* | 80 | 116.40 | 5.52 | 51.6 | 1697.61 | 61.32 | 12.12 | 73.44 |
| “NR0160305” | 4 g dm^-3^ | *Control* | 100 | 123.60 | 5.90 | 43.6 | 1656.02 | 59.36 | 16.78 | 76.14 |
| “NR0160305” | 4 g dm^-3^ | *Control* | 120 | 124.80 | 6.12 | 42.2 | 1457.19 | 58.18 | 18.54 | 76.72 |
| “NR0160305” | 0 g dm^-3^ | Tríppel^®^ | 0 | 29.60 | 2.10 | 19.0 | 189.85 | 33.04 | 8.46 | 41.50 |
| “NR0160305” | 0 g dm^-3^ | Tríppel^®^ | 20 | 50.36 | 2.86 | 22.0 | 374.51 | 36.20 | 8.02 | 44.22 |
| “NR0160305” | 0 g dm^-3^ | Tríppel^®^ | 40 | 60.36 | 3.05 | 27.8 | 483.03 | 28.60 | 7.04 | 35.64 |
| “NR0160305” | 0 g dm^-3^ | Tríppel^®^ | 60 | 60.80 | 3.39 | 34.0 | 619.94 | 28.62 | 6.10 | 34.72 |
| “NR0160305” | 0 g dm^-3^ | Tríppel^®^ | 80 | 61.30 | 3.62 | 26.0 | 508.91 | 26.02 | 5.04 | 31.06 |
| “NR0160305” | 0 g dm^-3^ | Tríppel^®^ | 100 | 61.60 | 3.86 | 21.2 | 379.78 | 18.54 | 4.10 | 22.64 |
| “NR0160305” | 0 g dm^-3^ | Tríppel^®^ | 120 | 61.84 | 3.89 | 15.0 | 218.37 | 17.96 | 3.22 | 21.18 |
| “NR0160305” | 4 g dm^-3^ | Tríppel^®^ | 0 | 31.20 | 2.18 | 20.8 | 210.86 | 33.78 | 9.02 | 42.80 |
| “NR0160305” | 4 g dm^-3^ | Tríppel^®^ | 20 | 54.80 | 3.14 | 25.6 | 652.63 | 39.22 | 13.04 | 52.26 |
| “NR0160305” | 4 g dm^-3^ | Tríppel^®^ | 40 | 83.36 | 4.08 | 36.8 | 1321.90 | 50.06 | 16.00 | 66.06 |
| “NR0160305” | 4 g dm^-3^ | Tríppel^®^ | 60 | 102.60 | 5.11 | 49.0 | 1745.14 | 53.72 | 15.58 | 69.30 |
| “NR0160305” | 4 g dm^-3^ | Tríppel^®^ | 80 | 124.40 | 5.91 | 54.4 | 2047.69 | 56.98 | 11.30 | 68.28 |
| “NR0160305” | 4 g dm^-3^ | Tríppel^®^ | 100 | 130.40 | 6.44 | 45.2 | 1739.43 | 60.54 | 16.18 | 76.72 |
| “NR0160305” | 4 g dm^-3^ | Tríppel^®^ | 120 | 131.00 | 6.61 | 36.2 | 1395.73 | 60.64 | 14.88 | 75.52 |
| “NR0160305” | 0 g dm^-3^ | Torpeno^®^ | 0 | 27.90 | 1.79 | 19.0 | 189.57 | 32.32 | 7.92 | 40.24 |
| “NR0160305” | 0 g dm^-3^ | Torpeno^®^ | 20 | 49.80 | 2.55 | 22.6 | 364.81 | 35.94 | 8.44 | 44.38 |
| “NR0160305” | 0 g dm^-3^ | Torpeno^®^ | 40 | 57.84 | 2.85 | 25.0 | 425.85 | 28.12 | 6.92 | 35.04 |
| “NR0160305” | 0 g dm^-3^ | Torpeno^®^ | 60 | 58.52 | 3.10 | 32.4 | 531.15 | 28.48 | 5.70 | 34.18 |
| “NR0160305” | 0 g dm^-3^ | Torpeno^®^ | 80 | 59.20 | 3.16 | 24.6 | 414.10 | 25.12 | 4.62 | 29.74 |
| “NR0160305” | 0 g dm^-3^ | Torpeno^®^ | 100 | 60.10 | 3.33 | 16.0 | 255.18 | 19.16 | 5.36 | 24.52 |
| “NR0160305” | 0 g dm^-3^ | Torpeno^®^ | 120 | 59.90 | 3.48 | 10.2 | 139.52 | 15.86 | 3.10 | 18.96 |
| “NR0160305” | 4 g dm^-3^ | Torpeno^®^ | 0 | 27.00 | 2.01 | 18.0 | 185.10 | 35.18 | 6.54 | 41.72 |
| “NR0160305” | 4 g dm^-3^ | Torpeno^®^ | 20 | 48.52 | 3.00 | 22.2 | 535.64 | 62.58 | 11.72 | 74.30 |
| “NR0160305” | 4 g dm^-3^ | Torpeno^®^ | 40 | 73.26 | 3.76 | 35.6 | 1134.16 | 56.24 | 18.58 | 74.82 |
| “NR0160305” | 4 g dm^-3^ | Torpeno^®^ | 60 | 86.60 | 4.80 | 41.6 | 1441.66 | 57.80 | 17.06 | 74.86 |
| “NR0160305” | 4 g dm^-3^ | Torpeno^®^ | 80 | 106.00 | 5.68 | 53.6 | 1953.91 | 58.10 | 17.52 | 75.62 |
| “NR0160305” | 4 g dm^-3^ | Torpeno^®^ | 100 | 116.40 | 6.18 | 47.0 | 1909.82 | 59.84 | 21.88 | 81.72 |
| “NR0160305” | 4 g dm^-3^ | Torpeno^®^ | 120 | 118.60 | 6.41 | 39.0 | 1602.83 | 59.10 | 18.46 | 77.56 |

^a^ Values represent the means of 5 replicates at each evaluation point. These data are the basis for the longitudinal trend graphs (Fig. 1a, 1b, 2a, 2b, 3a, 3b, and 3c). PH = Plant height (cm); SD = Stem diameter (mm); NL = Number of leaves (n); LA = Leaf area (cm^2^); Chl *a*, Chl *b*, Chl *a+b* = Chlorophyll indices (FCI); DAT = Days After Transplanting.
